# Linking New Alleles at the Oscillator Loci to Flowering and Expansion of Asian Rice

**DOI:** 10.3390/genes14112027

**Published:** 2023-10-31

**Authors:** Guangtong Gao, Maoxian Chen, Rong Mo, Nan Li, Yunzhang Xu, Yingqing Lu

**Affiliations:** 1 State Key Laboratory of Systematic and Evolutionary Botany, Institute of Botany, Chinese Academy of Sciences, 20 Nan Xin Cun, Beijing 100093, China; gaoguangtong@ibcas.ac.cn (G.G.); chenmaoxian@mail.kib.ac.cn (M.C.); linan90633@163.com (N.L.); xuyunzhang239@126.com (Y.X.); 2 University of Chinese Academy of Sciences, Beijing 100049, China; 3 State Key Laboratory of Plateau Ecology and Agriculture, Qinghai University, Xining 810016, China

**Keywords:** central oscillator, recombination, allelic expression, mutations, positive selection, negative selection, 5′ region, range expansion

## Abstract

The central oscillator is believed to be the key mechanism by which plants adapt to new environments. However, impacts from hybridization, the natural environment, and human selection have rarely been assessed on the oscillator of a crop. Here, from clearly identified alleles at oscillator loci (*OsCCA1/LHY*, *OsPRR95*, *OsPRR37*, *OsPRR59*, and *OsPRR1*) in ten diverse genomes of *Oryza sativa*, additional accessions, and functional analysis, we show that rice’s oscillator was rebuilt primarily by new alleles from recombining parental sequences and subsequent 5′ or/and coding mutations. New alleles may exhibit altered transcript levels from that of a parental allele and are transcribed variably among genetic backgrounds and natural environments in RIL lines. Plants carrying more expressed *OsCCA1_a* and less transcribed *OsPRR1_e* flower early in the paddy field. 5′ mutations are instrumental in varied transcription, as shown by EMSA tests on one deletion at the 5′ region of highly transcribed *OsPRR1_a*. Compared to relatively balanced mutations at oscillator loci of *Arabidopsis thaliana*, 5′ mutations of *OsPRR37* (and *OsCCA1* to a less degree) were under negative selection while those of *OsPRR1* alleles were under strong positive selection. Together, range expansion of Asian rice can be elucidated by human selection on *OsPRR1* alleles via local flowering time-yield relationships.

## 1. Introduction

Although nucleotide sequences have reached an astronomical scale, knowledge on their connections to enchanted phenotypes of nature is still at the stage of infancy. Systematic efforts focusing on human [1,2], mouse [3,4,5], *Caenorhabditis elegans* [6,7], yeast [8,9], *Arabidopsis* [10,11,12], and apple [13] in recent years, for instance, partly illustrate how much progress has been made. Since a tremendous amount of work is still ahead, it is critical to reflect what can be done to improve future predictions of relationships between genes and phenotype. As a major feature of genes, genetic polymorphisms have been described in several ways [14], all of which require comparisons of at least two homologous sequences to show the existence of different morphs. In recent literature, three describers of genetic polymorphism are frequently encountered: the single nucleotide polymorphism (SNP), haplotype, and allele. SNPs refer to substitutions seen in two or more sequences, which are context-specific and easy to obtain, but not a fixed property of a sequence or genome. Haplotype, on the other hand, is a haploid sequence *per se*, which can maintain information of gene arrangement on the same chromatid/genome. Relative to these terms, the term allele is decades older, designating a functional form of a gene in a population [15]. It was defined only conceptually in the early days, until it was proposed to use “defining mutations” as criteria [16]; still, the number or/and locations of the mutations have not been elaborated. Earlier studies of allelic series focused primarily on the coding regions [17,18]. The whole-gene assays on allelic series appeared relatively later [19,20], along with advancing of the genome era. Considering that the allele is the acting gene in each individual, it is naturally the basic unit for exploring gene-phenotype relationships.

A broad comparison of allelic impacts on phenotype requires a practical definition of a biological allele, the length of which is closer to that of the average gene in a genome. Over the last decades, patterns of nucleosome occupation [21] and DNase I signal [22] across multiple genes suggest that transcriptional activities of most genes take place within the approximately 1 Kb region upstream of the translation starting site, and most transcript isoforms at a 3′ region appear within 100 bp after the stop codon in yeast [23]. To enable a practical and consistent usage of the concept of allele, we have adopted the following working definition of an allele: it is a functional unit of a gene, including a genomic sequence that contains a 5′-region (typically at least 1 Kb), coding region (s) with or without intron(s), and a 3′-region (at least 100 bp). The exact range of a specific allele, when a study requires such information, can be always experimentally determined as seen in functional analysis. Since an allele frequently segregates with other alleles at the same locus in a population, it is recognizable by its recurring appearance in more than one individual. Except in few cases [17], little is known about how a new allele emerges in a biological system after hybridization or speciation. The goal of this research was to perform an allelic analysis on a critical molecular system, based on above allelic definition, in order to understand emergence of new alleles after a divergent event and detect possible connections between allelic polymorphism and phenotype in a natural environment.

This quest was made possible in Asian rice (*Oryza sativa* L.), one of the crops capable of generating recombinant inbred lines (RILs). These lines have greatly reduced heterozygosity, facilitating a more reliable identification of allelic impacts on phenotypes when carriers of the relevant alleles grow in the same environment. The design is to use high-generation RILs (F5 and beyond) and requires identification of alleles in repeatable and verifiable ways, which will hopefully lead to a better description of context-specific allelic function and allelic connection to phenotype. Specifically, we explored here formations of alleles of oscillator genes after hybridization and selection in Asian rice and allelic effects on phenotypes including flowering days and yield.

Asian rice has been shown originating directly from hybridization between perennial *Oryza rufipogon* (*Or*) and annual *O*. *nivara* (*On*) [20], providing an ideal system for allelic analysis to address issues such as to what extent recombination has changed the central oscillator (oscillator) and how domestication altered the oscillator system during range expansion of rice. Following early characterizations of oscillator genes and their products of *O. sativa* (*Os*) [24,25,26], more impacts of the clock on rice have been documented in recent years. For instance, OsPRR37 (an ortholog of PSEUDO-RESPONSE REGULATOR 7 (PRR7) of *Arabidopsis thaliana* (L.) Heynh) suppresses flowering under natural long-day condition, but can be an activator/suppressor under natural short-day condition, depending on interactions among flowering regulators [27] including Hd1 [28], Ghd7 [29], and DTH8 [30], thus considered part of the Hd1-DTH8-Ghd7-PRR37 module for photoperiodic sensing of flowering [31]. OsPRR59 (an ortholog of AtPRR5) can repress flowering via direct binding to the promoter of *Ehd3* [32], a flowering-promoting gene in rice [33]. Other phenotypes can be influenced by oscillator genes as well. OsPRR59 and OsPRR95 (an ortholog of AtPRR9) can suppress expression of a gene (*OsMGT3*) that encodes a transporter of magnesium in chloroplasts [34]. Unlike *Arabidopsis* CIRCADIAN CLOCK ASSOCIATED1 (CCA1) and LATE ELONGATED HYPOCOTYL (LHY) [35,36], which interact and are partially redundant, only one gene, *OsCCA1*/*OsLHY* exists as their ortholog in Asian rice. The gene has two haplotypes (indica and japonica) showing different effects on flowering time [37]. Through sugar sensing and strigolactone signaling, enhanced expression of *OsCCA1* may repress tiller-bud and panicle growth whereas increasing transcription of *OsPRR1* has the opposite effects [38]. OsCCA1 can regulate signaling of abscisic acid and potentially participate in the tolerance mechanism of abiotic stress [39]. Most studies so far have focused on molecular connection of oscillator loci with genes in other pathways/phenotypes, which is significant but can rarely be translated into allelic impacts per se to guide breeding practice. Allelic expression level is a significant parameter in evaluation of allelic impacts; nonetheless, natural expressions of oscillator genes have rarely been systematically documented in rice [40,41], and quantitative effects of alleles are even rarer to be assessed in a natural environment.

Impact of selection on oscillators is also less known. Human domestication is an escalating process for mutation accumulation due to intense collections by human on new variants. Its magnitude relative to that of natural selection has seldom been studied. To compare impacts of human selection with those of natural selection, we identified allelic series of key genes of the oscillator in *A*. *thaliana* (*At*). Much of the pioneering work on plant oscillators has been conducted in the plant, including sequentially peaked mRNAs from morning to evening of *CCA1*, *PRR9*, *PRR7*, *PRR5*, and *PRR1* [42,43,44,45], and about 2 h delay of protein peak from the mRNA peak shown by PRR5 quantified under 12 h light/12 h dark condition [46]. *AtCCA1* can respond to phytochrome-mediated light signaling to be activated [47] and monitor transcript levels of PRRs genes such as *PRR5* [48], which are generally considered transcriptional repressors [49], by repressing their transcriptions [50]. Further, *AtTOC1* (TIMING OF CAB EXPRESSION 1, or *AtPRR1*) may directly respond to light intensity, and its mutant, *toc1*-*1*, shows a 2–3 h shorter period and diminished response to day-length at flowering [51,52]. Consistently, increasing *AtTOC1* dosage may delay clock pace and enhance light sensitivity [53]. PRRs may prolong the stability of CONSTANS (CO), a positive activator of FLOWERING LOCUS T, under a long-day condition to promote flowering in *Arabidopsis* [54]. Numerous cellular activities are under the influence of the oscillator genes [55,56]. For instance, AtPRR9 may influence leaf senescence by promoting aging-related positive regulator ORESARA1 [57]. AtPRR7 and AtPRR5 may bind to PHYTOCHROME-INTERACTING FACTORs (PIFs) to repress the target genes of PIFs [58].

Here, we focus on oscillator-flowering/fruiting relationships. By engaging genomic and functional analyses, we search for cases showing how a new allele emerges in this critical biological system and allelic impacts on phenotypes of Asian rice. Besides sequence analysis of alleles, allelic expression patterns in a natural environment were also documented, along with flowering times and individual fruit sets in RIL populations. For allelic functions, we explored factors influencing allelic transcription using both laboratory and field data and tested functional alleles of Asian rice in mutants of *A. thaliana*. Impacts of human selection were inferred via comparisons of allelic diversities across circadian clock genes between species and geographic distributions of alleles at oscillator loci of Asian rice. In light of new functional data and past history of human selection, impacts of oscillators on flowering and yield can be better understood at allelic level in Asian rice.

## 2. Materials and Methods

### 2.1. Assessing Allelic Diversity across Oscillator Loci

#### 2.1.1. Defining Alleles from Genomic Data

Either 5′ differences or amino-acid differences (or both in cases) were taken to define an allele, unless they are proven not causing a functional change of the gene. In real data, a class of changes is frequently seen at 5′ regions, which is a string (>3 bp) of one nucleotide, such as A-string, C-string, etc. These strings are prone to sequencing errors and can be misleading if used as the sole criterion for a new allele. In these cases, we search for at least one additional change that is independent or involves no strings before assigning a new allele, for the purpose of abating possible errors. Intron-only changes are not taken as the sole criterion for defining a new allele unless it is proven that the differences may lead to a functional alteration of the gene. To acknowledge such variants, sequences with intron-only or within-allele changes are referred to as allelets. No further analysis was performed on these allelets in this study. Though taken as neutral variants, allelets can be either transient or fixed.

#### 2.1.2. Analysis of Ten Diverse Nuclear Genomes of *O. sativa*

Applying the definitions above, we conducted a genetic survey of alleles at the oscillator loci of *O. sativa* (*Os*) based on high-quality genomes, using genomes (PRJNA48107, PRJEB4137) of its parental species *O*. *rufipogon* and *O*. *nivara* as references. Nuclear genomes were chosen for their sequencing techniques, chromosome-level assembly, and high coverage [20,59] and downloaded from the NCBI database, as described [60]. Sequences of 10–20 Kb around each of the five oscillator genes were sliced from the genomes and aligned according to genic regions. The parental orthologs were first compared to each other to make sure that enough genetic polymorphisms were available at each locus for later analysis. Mutations specific to Asian rice were subsequently identified at each locus, as shown previously [20]. As a useful feature of locus, mutation density (number of mutations per nucleotide per locus per period of comparison) was calculated for 5′ and coding regions of each gene. It was then compared to that of baseline sequences located in the neighborhood of the locus in the same set of genomes. A baseline sequence is used to detect background mutations, which cannot be from regions with annotated functions (in order to avoid a possible distortion on the background mutation rate). It is a neutral sequence between genes (intergenic region) or a long intron (>800 bp) within the locus (to be comparable to the length of 5′ region or coding regions), as detailed below.

#### 2.1.3. Tests of Selection

Since mutations here can be localized to specific alleles, tests of selections can be either allele-specific using the ratio of Ka/Ks or d_N_/d_S_ [61,62] when both synonymous and nonsynonymous mutations are present, or locus/allele-specific by comparing the average mutation rate of alleles to that of their co-genomic neutral regions (referred to as baseline test here). The former test can be carried out by obtaining numbers of synonymous sites and nonsynonymous sites for each allele in DNAsp [63] and computing Ka (number of nonsynonymous mutations per nonsynonymous site) and Ks (number of synonymous mutations per synonymous site) for each allele based on the specified classes of mutations. For the latter test, a baseline sequence can be a long intron within the tested gene or a neutral genomic region downstream of 3′ of each gene or upstream of 5′ of the gene, whichever is closer and appropriate. When the next annotated feature to the tested gene is far and the intergenic region is near (>800 bp) or longer than 1 Kb, it can serve as a sample of the baseline reference. When the gene region is clustered with annotated features, the further next intergenic region can be considered, as in the case of *OsPRR59* here. Having the baseline sequence close to a tested gene also ensures the minimum impact of regional variation of background mutation rate on the test. Allelic mutation rate significantly higher than the background mutation rate indicates positive selection and the opposite indicates negative selection.

#### 2.1.4. Additional Surveys of Varieties of *O. sativa* to Validate Allelic Sequences

Since mutation analysis is highly sensitive to sequencing errors, which happen largely randomly, we evaluated the accuracy of the alleles by sequencing additional accessions of Asian rice at mainly 5′ and coding regions of three loci (*OsPRR1*, *OsPRR37*, and *OsCCA1*) using Sanger’s technique. This may lead to finding unreported alleles.

#### 2.1.5. Parallel Analysis on Ten Nuclear Genomes of *A. thaliana*

Since *A*. *thaliana* distributes over a range of latitudes that are comparable to those of Asian rice and many of its ecotypes have been sequenced at different levels [10], its nuclear genomes at public databases can be found with coverage larger than 80×. We sampled ten of these genomes according to sequencing quality and carriers’ distribution. To estimate mutation number, we took an approximate method by initially assuming that a polymorphic site in an alignment of co-specific sequences (≥10) occurs in less than 30% of the ecotypes being from a recent mutation and then check the accuracy of the results with the genome of an outgroup, *A*. *arenosa* (GCA_026151155.1), using the parsimony principle. We identified tentative alleles at each of five oscillator loci (*AtCCA1*, *AtPRR9*, *AtPRR7*, *AtPRR5*, and *AtPRR1*). Though the parental species of *A. thaliana* is not known, its con-generic species, *A*. *arenosa*, has been sequenced at the genome level [64] and may share ancestral sites with the immediate ancestor and *A. thaliana* whereas novel mutations are specific to *A*. *thaliana*. Some of the tentative mutations identified in *A*. *thaliana* were excluded if they also appeared in the genome of *A*. *arenosa* (*Aa*), as revealed by alignments of orthologous sequences. An error rate was estimated based on the comparisons. Though the protocol may still carry an error rate larger than those of mutations identified in *O*. *sativa*, comparisons of patterns between species sampled in the same size are still informative. For a functional analysis on mutations, a rigid validation process of the mutations is needed via additional sequencing.

### 2.2. Variation of Allelic Expressions in the Paddy Field

#### 2.2.1. Preparation of Populations from RILs

For functional analysis on mutations in Asian rice, two landraces, Heidao (as maternal parent) and Jixuenuo (as paternal parent), were crossed twice at Hainan (China), giving rise to two RIL lines (line298 and line315 from F5 to F8) tested later at a paddy field of IBCAS. Heidao was from a northern province (Heilongjiang), and Jixuenuo was from Yunnan, a southwest province of China. At F5 generation in 2017, three *OsPRR37* alleles (*OsPRR37*_*h*, *OsPRR37*_*b*, and *OsPRR37*_*purp3*) were homozygous in six populations. At F6 generation, line315 had four populations homozygous for *OsPRR1*_*d* and one for *OsPRR1*_*b* (but *OsCCA1* and *OsPRR37* varied) and line298 has two populations homozygous for *OsPRR1*_*b*. At F7 generation in 2019, line315 had one population homozygous for *OsPRR1*_*a*, *OsCCA1*_*a*, and *OsPRR37*_*h*, and line298 had one homozygous for *OsPRR1*_*e*, *OsCCA1*_*d*, and *OsPRR37*_*purp3*. At F8 generation in 2020, population D5 of line315 was homozygous at *OsPRR1* (*OsPRR1*_*d*) and *OsPRR37* (*OsPRR37*_*h*) but segregating at *OsCCA1* (*OsCCA1*_*a* and _*c*), permitting more accurate estimations of allelic expressions of *OsCCA1*. In line298, three populations were identical in *OsPRR1* (*OsPRR1*_*e*) and *OsPRR37* (*OsPRR37*_*h*) but one was homozygous for *OsCCA1*_*a* (D3) and the other two were for *OsCCA1*_*c*, allowing comparison of flowering times between alleles *OsCCA1*_*a* and *OsCCA1*_*c*. Populations from the third RIL line309 (from Bosanger (landrace) × Yunjin85 (variety), both from Yunnan) and fourth RIL line301 (from Bosanger (landrace) × IR661-1 (variety)) were also prepared and used as Supplemental Materials. All allelic information is shown in the result section.

#### 2.2.2. Field Transcriptions of *OsCCA1*, *OsPRR1*, and *OsPRR37* in a 48-h Interlude

During the summer of 2019, fresh leaf segments of two plants were sampled with a nitrogen tank at 2 h intervals from each of two populations homozygous for *OsPRR1*_*b* (one from line298 and the other from line309) between 10 a.m. of 14 August and 8 a.m. of 16 August in the paddy field. The samples were then transferred to −80 °C before further processing. RNAs were extracted from each of the samples and reversely transcribed into the first-strain cDNAs and quantified with the Picogreen protocol. The standard sequences of the alleles were inserted into pEASY-vectors and quantified to be used as internal references. All samples were processed via the same absolute quantification method [65] to allow comparisons within and between experiments.

Similarly, during the summer of 2020, fresh leaf segments of four individuals of D5 (two homozygous for *OsCCA1*_*a* and two for *OsCCA1*_*c*) and one (homozygous for *OsCCA1*_*a*) of D3 were sampled at 2 h intervals between 10 a.m. of 13 August and 8 a.m. of 15 August and processed as described above. While transcript levels of *OsCCA1* alleles were estimated in three plant series, those of *OsPRR37* (*OsPRR37*_*h* and *OsPRR37*_*b*) and *OsPRR1* (*OsPRR1*_*e* and *OsPRR1*_*g*) were measured in two plant series (one for each allele).

#### 2.2.3. Fixed-Time Transcriptions of OsCCA1 and OsPRR1 over Six Days Prior to Flowering

From F6 generation in 2018, we sampled homozygous populations of *OsPRR1* (*OsPRR1*_*d* and *OsPRR1*_*b*) in the evening between 6 p.m. to 8 p.m. over six consecutive days (4–9 August) to reduce environmental influence. The sampling period was further narrowed down to about a half hour in 2019 and 2020 to improve accuracy of estimation. All samples were taken from the flag leaves at the same position (about 2–4 cm from the tip of leaf) and frozen in liquid nitrogen immediately before transferring to −80 °C. Accessions homozygous for *OsCCA1* were sampled in the early morning (starting at 6 a.m.) and those for *OsPRR1* sampled in the evening (6:30–7:00 p.m.) in 2019. The weather conditions were noted at the sampling site and daily extremes from the local meteorological records.

### 2.3. Functional Analysis on Mutations/Alleles of Oscillator Genes

#### 2.3.1. Effects of Natural Temperature and Genetic Background on Allelic Expression

Daily transcript levels of alleles were compared against temperature at time of sampling and the high, low, or average temperatures of the days across loci using Spearman rank correlation coefficients. Effect of genetic background was evaluated within an RIL population, which had one of the oscillator loci segregating with two alleles but the targeting locus homozygous.

#### 2.3.2. Effect of 5′-Deletion in OsPRR1 on Binding Capacity of OsCCA1 via Electrophoretic Mobility Shift Assays (EMSA)

The DNA binding domain of OsCCA1 was inferred from the alignments of orthologs across species of *Oryza*, *Arabidopsis*, and *Ipomoea*. The sequence was amplified with primers (OsCCA1EcoRIf: 5′ CCGGAATTCATGGAGATTAATTCCTCTGGTGAG 3′ and OsCCA1SalIr: 5′ ACGCGTCGACTGCCATTTGTGCAGTGCTATTG 3′) from cDNAs of Heidao, inserted into pCold vector (Takara), and expressed in *E*. *coli* strain Transetta (DE3) for protein expression. The expressed protein was harvested via a column of Ni Sepharose (GE Healthcare, Boston, MA, USA); its concentration was estimated, and it was stored at −80 °C. Probes containing segments of the promoter sequences of *OsPRR1*_*a* and *OsPRR1*_*e* were made from pairing complementary oligo-nucleotides at room temperature. Reaction of DNA-protein binding was performed in 10 µL volume with 2 µL 5× binding buffer (Invitrogen, ThermoFisher, Waltham, MA, USA), 1.3 µg protein, and 20 pmol probe mixed at 23 °C for 20 min. Next, 8 µL of the resulting solution was loaded to a 10% non-denaturing polyacrylamide gel for electrophoresis (100 V, ~70 min). The gel was treated and photographed with the previous settings described in Wang et al. [66].

#### 2.3.3. Compatibility Tests of *Os* Alleles in Mutants (*cca1-1* and *toc1-1*) of *A. thaliana*

Divergences of *PRR1* and *CCA1* were assessed by introducing 5′ and coding sequences of *Os* alleles into mutants (*toc1-1* and *cca1-1*) of *A. thaliana*, respectively. The sequences were inserted into pCAMBIA1301 vectors, which were introduced into *Agrobacteria tumefaciens* to infect the mutants at flowering stages. For *OsCCA1* alleles, T1 generation of a transformed mutant was examined for length of hypocotyl and days to flowering, and T2 generation was examined for accumulation of anthocyanins. For *OsPRR1* allele, phenotypes were all collected on the T2 generation.

Measurements of hypocotyls were taken under a microscope (Leica DVM6, Leica Microsystems, Wetzlar, Germany) from 6-day-old seedlings growing in a growth chamber under the condition of 22 °C 16 h light/20 °C 8 h dark, and counting of flowering days started from seed germination on petri dish with MS medium. For transformants of *OsCCA1*, anthocyanin content was measured from 3-day-old seedlings (~0.025 g extracted in methanol with 1% HCL (*w*/*v*), overnight at 4 °C); the solution was mixed with the same quantity of chloroform and the supernatant was kept. Absorption of the clean supernatant was taken under 530 nm and 657 nm using a spectrophotometer (Evolution, ThermoFisher, Waltham, MA, USA), and the difference (A_530_–A_657_) was used to estimate anthocyanin content, as described previously [67].

For *OsPRR1*, measurements of anthocyanins were from plants grown for 10 days and then treated at 10 °C for one week before harvested with roots removed. The plants (100 mg) were extracted in 1 mL freshly made solution of butanol:HCl:water (18:1:81). After boiling for 3 min, the solution was incubated at 25 °C for 24 h and then centrifuged for 40 min. The supernatant was measured for absorbance under 535 nm and then 650 nm. The anthocyanin content was estimated by (A_535_–2.2 A_650_) as described previously [68].

### 2.4. Identifying Alleles Associated with Flowering Times and Yields

For allelic impact of *OsPRR37* on flowering time, six RIL populations, all of which were F5, were tested in 2017 summer in the paddy field at IBCAS. Six accessions (two homozygous for each of *OsPRR37*_*h*, *OsPRR37*_*b*, and *OsPRR37*_*purp3*) were completely sampled over the flowering season. For allelic impacts of *OsPRR1* and *OsCCA1*, samples were taken from the earliest and latest flowering individuals (2 of each type, all from non-border plants) per accession over multiple accessions, of which the alleles had been identified by sequencing. The plants were then harvested at the end of growth season for total panicles per plant, which were air-dried and weighted to 0.001 g. All accessions used in each experiment were treated equally during growth. Each accession was grown at a density of about 36/m^2^, starting from early May and ending in later October.

### 2.5. Distributions of Oscillator Alleles in Traditional Cultivation Regions of O. sativa

The primary location of each genome, landrace, or variety was from records or publications. The alleles of oscillator genes follow their carriers in distribution. When an allele appeared in more than one source in different regions, all relevant regions were mapped.

### 2.6. Statistical Analysis

Comparisons of means were mostly carried out by student’s *t*-tests, which can be performed within Excel; for large samples, a *z*-test was conducted instead. For a non-parametric test on correlation, Spearman’s correlation coefficient (r_s_) was tested under H_0_: r_s_ = 0 against critical values of the table A31 [69]. When a relationship was fit for data between x and y variables, it was evaluated by the generalized least-square technique implemented in R-protocols (nlme). The residuals were examined for normality and the confidence intervals around parameters were estimated.

## 3. Results

### 3.1. Rebuild of Circadian Clock Revealed by Allelic Diversities at Oscillator Loci of O. sativa

#### 3.1.1. Genomic Survey of Five Oscillator Genes

**Figure 1:**
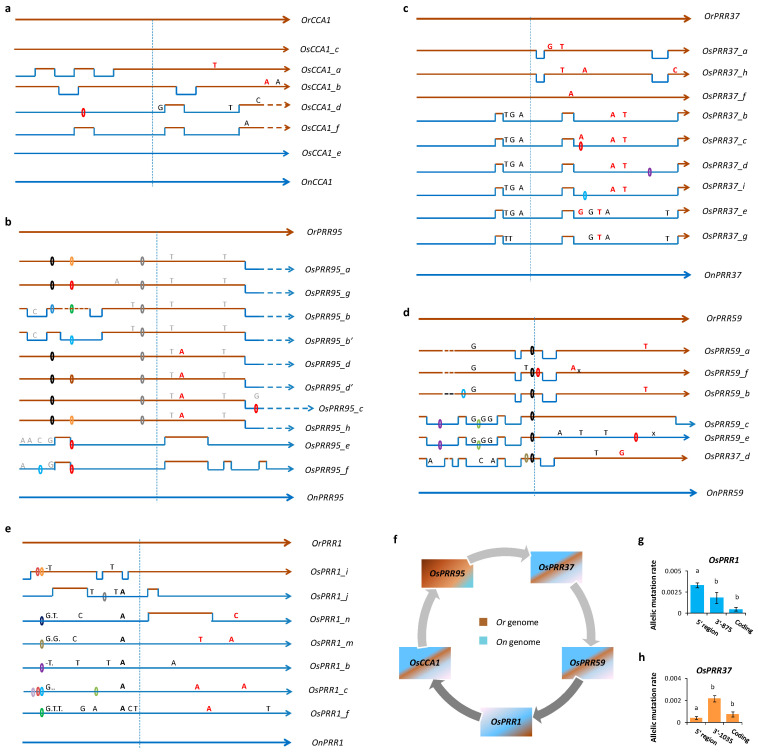
Features of alleles at five oscillator loci in ten diverse genomes of *O. sativa*. (**a**) Six alleles of *OsCCA1*. Each allele begins from about 1 Kb of 5′ region and then the entire coding regions (with introns excluded), as shown by the arrowed line (5′→3′). The vertically dashed line indicates the start codon for the coding regions to the right. The sequence identical to *OrCCA1* is in brown color, and that identical to *OnCCA1* in blue. The recombination events (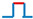) were estimated from neighboring polymorphic sites, with colors showing origins of parental sequences. Regions of uncertain origin are shown in horizontally dashed lines. *Os*-specific mutations are shown by a circle for indel (colors for different ones) or letter for substitution (red for the nonsynonymous and black for the synon-ymous). The arrow ends at the stop codon of each allele, with the allele designated in lower letter following the locus. Drawings are not proportional to the genic regions but show relative positions of features. (**b**) Eight alleles of *OsPRR95*. Two tentative allelets (*OsPRR95*_*b*′ and _*d*′) are included here. Different indels are shown in colors. (**c**) Nine alleles of *OsPRR37*. (**d**) Six alleles of *OsPRR59*. An early stop codon caused by mutation is shown by x. (**e**) Seven alleles of *OsPRR1*. (**f**) Relative con-tributions of parental genomes to the oscillator loci in Asian rice. The components of the molecular clock are in square with parental origins of their sequences in colors. Arrows indicate flow of time, with dark ones for the night. (**g**) Allelic mutation rates across genic regions of *OsPRR1*. Seven alleles shown in **e** are included. The baseline (3′-875) is from 875-bp genomic region downstream of the 3′ region. Letters a and b indicate a significant *t*-test after correction for multiple comparisons at ex-perimental error rate of 0.05. See Table 2 for detail. (**h**) Allelic mutation rates across genic regions of *OsPRR37*. Nine alleles (shown in (**c**)) are included. The baseline (3′-1035) is from 1035-bp genomic region downstream of the 3′ region. Format of tests follows that of (**g**).

*CCA1*, *PRR95*, *PRR37*, *PRR59*, and *PRR1* were identified from nuclear genomes of *O*. *nivara* and *O*. *rufipogon* based on known *Os* sequences. A well-diverged pattern between the species (Table S1) allows identifications of parental contributions to their hybrid offspring, *O*. *sativa*, with sufficient markers. Comparisons of the *Os* sequences with parental orthologs suggest that recombinants were present at all oscillator loci, involving 5′ or/and coding regions (Figure 1). Ten diverse nuclear genomes of *O*. *sativa* interrogated here cover all five subgroups recognized so far, including *aus* (Netal Boro: PRJNA565483 and N22: PRJNA315689), *tropical japonica* (Ketan Nangka: PRJNA564615 and Chao Meo: PRJNA565484), *japonica* (Nipponbare: PRJNA12269 and Kitaake: PRJNA448171), *indica* (9311: PRJNA427873, Shuhui498: PRJNA318714, and Minghui63: PRJNA30254), and *aromatic* (IRGC 12485-1: PRJNA565479a). Using the allelic definition introduced above, we identified 6 to 9 alleles at each of the oscillator loci (Table 1). After the initial hybridization of Asian rice, the newly formed recombinant alleles segregate sometimes with ancestral alleles that directly passed down without changes (Figure 1). Hereafter, we refer to the latter as parental alleles.

For *OsCCA1*, six alleles are present in the ten genomes (Figure 1a); four of them (*OsCCA1*_*a*, _*b*, _*d*, and _*f*) are recombinants and two are parental alleles (*OsCCA1*_*c* from *O*. *rufipogon* and *OsCCA1*_*e* from *O*. *nivara*). Although *OsCCA1_d*, *_e*, and _*f* encode the same protein, *OsCCA1_d* and *OsCCA1* _*f* were derived from different recombination events at the 5′ regions, with three synonymous mutations accumulated in *OsCCA1_d*. Overall, eight mutations occurred to the four recombinant alleles of *OsCCA1* (Table 1 and Table S2).

For *OsPRR95*, eight recombinant alleles (*OsPRR95_a*, *_b*, *_c*, *_d*, *_e*, *_f*, *_g*, and *_h*) were identified in the *Os* genomes (Figure 1b), involving five recombination patterns and twenty *Os* mutations (Table S3). Among them, *OsPRR95_b* has an allelic variant (*OsPRR95_b*′) that shows only differences in A- and C-strings at the 5′ region, and *OsPRR95_d* allele has a variant (*OsPRR95_d*’) of one indel in an A-string at the 5′ region. Both variants are taken tentatively as allelets here. *OsPRR95_c* has the same 5′ region as *OsPRR95_d* but encodes a bigger protein due to an indel towards the end of the coding regions, which delayed the stop codon (Table S3). The most unique allele is *OsPRR95_h* from *aromatic*, which appears to be a recombinant of *OsPRR95_a* and *OsPRR95_d*, possessing the *5′* region of the former and the coding region of the latter without further mutations (Figure 1b). This pattern confirms the hybrid origin of the subgroup *aromatic* recently reported [60].

At *OsPRR37*, nine alleles were recognized (Figure 1c). They are primarily recombinants, having 19 *Os* mutations accumulated in total. Interestingly, only two types of promoters are present. One is from the 5′ region of *OrPRR37* without change, used by *OsPRR37_a* and *OsPRR37_f* in *japonica*; the other is from the 5′ region of *OnPRR37* with three early mutations, used by alleles in *indica* including *OsPRR37_b*, *_c*, *_d*, _*e*, and _*i*. Coding regions also show two patterns of recombination; one is *Or*-biased, as shown at *OsPRR37_a*, *_f*, and *OsPRR37_h*, and the other is *On*-biased, as seen in alleles of *OsPRR37_b* from Shuhui498 and *aus* N22, *OsPRR37_c* from *tropical japonica* Chao Meo (CM), and *OsPRR37_d* from 9311, and *OsPRR37_i* from Minghui63 (Table S4). Three alleles, *OsPRR37 _c*, *OsPRR37*_*i*, and *OsPRR37*_*d*, are nonfunctional due to an indel amid their coding regions, which leads to a product of 304 amino acids encoded by *OsPRR37_c*, 313 amino acids by *OsPRR37_i*, or 509 amino acids by *OsPRR37*_*d*, instead of a protein of 742 amino acids, as encoded by the standard *OsPRR37_b* (Table S4). The nonfunctional alleles all lack the DNA-binding domain at the C-terminal.

The locus *OsPRR59* contains six alleles in the genomes surveyed, which generate only five distinct proteins, nevertheless, due to the same coding regions of *OsPRR59_a* and *OsPRR59_b*. These two alleles have largely different 5′ regions, with multiple deletions occurring to *OsPRR59_b* (Figure 1d). In contrast, two alleles, *OsPRR59_e* (from Minghui63) and *OsPRR59_f* (from *aus* Natel), are nonfunctional, as *OsPRR59_e* lacks the DNA-binding domain and *OsPRR59_f* misses additional domains. Since parental orthologs of PRR59 are not equal in length, allele *OsPRR59_c* inherited the C-terminal part of *OnPRR59*, encoding a protein 17 amino-acid longer than those given by the functional alleles *OsPRR59_a* and *OsPRR59_d.* The six alleles have accumulated 24 mutations in total (Table S5).

At *OsPRR1*, seven alleles (*OsPRR1_b*, *_c*, *_f*, *_i*, *_j*, *_m*, and *_n*) are present in the genomes (Figure 1e). *OrPRR1* has its entire coding (but not 5′) regions passed down to *OsPRR1_i* in *tropical japonica* (CM type), whereas *OnPRR1* was similarly inherited by several alleles (*OsPRR1_b*, *_c*, *_f*, and *_m*) with different levels of mutations accumulated, particularly at the 5′ region (Table S6). These alleles collectively gathered 36 mutations.

The allelic features above indicate a significant rebuild of the oscillator in Asian rice. While recombinant alleles took the central stage during the rebuild, genomic contributions from *O*. *rufipogon* and *O*. *nivara* differed among loci. *OsCCA1*, *OsPRR37*, and *OsPRR59* have more or less balanced contributions from parental genomes; alleles of *OsPRR95* are more *Or*-biased and those of *OsPRR1* that tend to be *On*-biased (Figure 1f). Since mutation density (including indels and substitutions but regardless of alleles) can characterize the mutation pattern of a locus [60], we compared this measure among the five oscillator loci and documented the highest mutation density at the 5′ region of *OsPRR1* and the lowest at the 5′ of *OsCCA1* (Table 1). The locus having the richest number of alleles is *OsPRR37*. These three loci were further investigated in the following experiments to understand how mutations affected phenotypes of Asian rice.

#### 3.1.2. Validations of Alleles at Loci of *OsCCA1*, *OsPRR37*, and *OsPRR1*

To ensure the highest accuracy of alleles across loci of oscillator genes for functional analysis, we sequenced three genes (*OsCCA1*, *OsPRR37*, and *OsPRR1*) using Sanger’s technique across multiple accessions that are independent from the analyzed genomes above. For *OsCCA1*, 28 plants from 20 accessions confirmed three alleles (*OsCCA1_a*, *OsCCA1_c*, and *OsCCA1_d*) at both 5′ and coding regions (Table S7). For *OsPRR37*, 23 individuals of 20 accessions were sampled, and two alleles (*OsPRR37*_*h* and *OsPRR37*_*b*) were validated (Table S7), which were previously known as *PRR37-2a* from H143 [70] and *OsPRR37-13* [71], respectively. The third allele, *OsPRR37*_*purp3*, which was absent in the ten genomes above, is identical to *OrPRR37* at both 5′ and coding regions. At the locus of *OsPRR1*, seven alleles (*OsPRR1_a*, *OsPRR1_b*, *OsPRR1_c*, *OsPRR1_d*, *OsPRR1_e*, *OsPRR1_g*, and *OsPRR1_h*) were recognized in 33 individuals of 20 accessions (Table S7), two (*OsPRR1_b* and *OsPRR1_c*) of which were identical to the sequences obtained from the genomes above. Five alleles (*OsPRR1_a*, *OsPRR1_d*, *OsPRR1_e*, *OsPRR1_g*, and *OsPRR1_h*) are absent in the ten genomes but present in other varieties/landraces.

Collectively, eight genome-based alleles were verified in the independent accessions and no errors were found in their sequences. In addition, one allele of *OsPRR37* (*OsPRR37*_*purp3*) and five alleles of *OsPRR1* are newly reported here (Table S8). Parental alleles are present at *OsCCA1* and *OsPRR37* in whole or in part, with the 5′ region of *OrPRR37* inherited by two japonica alleles (*OsPRR37_a* and *OsPRR37_f*) without change. Meanwhile, the coding regions of *OnPRR1* were passed on to an *indica* allele (*OsPRR1_a*) without changes (Figure S1).

### 3.2. Tests of Locus-Specific Selection

The five oscillator loci in Asian rice show three types of parental impacts—*Or*-biased, *On*-biased, or similar contributions of the parental genomes (Figure 1f). If a specific type of origin brings desired fitness either in a natural environment or under human selection, it can be favored. To detect positive selection at coding regions, we carried out d_N_/d_S_ test when synonymous and nonsynonymous mutations are both present within an allele, which led to a detection of positive selection at the coding regions of *OsPRR1*_*d* (d_N_/d_S_ = 1.2). For detection of selection among alleles not having sufficient substitutions for the above test or in genic regions such 5′ (which is excluded in the above test as well), we used the baseline test. It requires non-selected co-genomic sequence as baseline reference for each allele, which can be found in the sampled genome (Figure S2). For example, a fragment of 875 bp downstream of the 3′ region of an *OsPRR1* allele can be used as a baseline reference, and its average mutation number per nucleotide in the ten genomes can be compared to 5′ or coding regions of the *OsPRR1* alleles in the same genomes (Figure 1g). The period for the comparison is since the most recent common ancestor of the ten genomes till now, essentially covering the whole domestication history of Asian rice. Results indicate that among all five loci, only 5′ regions of *OsPRR1* have a mutation rate higher than the inter-genic region (one-tailed *t*-test, *p* = 0.0085), which indicates positive selection (Table 2). The result is significant after a correction for multiple comparisons (critical α’ = 0.0169, based on the Dunn-Šidák method [72]). The baseline test can also be applied to a single allele when the mean background mutation rate and its variation are available. Here, the mean background mutation rate per sequence of *OsPRR1* is 0.0003 (s.e. 0.000096), and it is significantly lower than allelic mutation rate of the coding regions (0.0032) or 5′ (0.0031) of *OsPRR1*_*d* (*t*-tests, *df* = 6, *p* < 0.001 in both cases), which is in agreement with the detection of positive selection above using the d_N_/d_S_ ratio.

In comparison, 5′ regions of *OsPRR37* underwent significantly negative selection (Figure 1h), causing the mutation rate to be significantly lower than those of not only the background but also coding regions (Table 2).

### 3.3. Functional Analyses on OsCCA1, OsPRR37, and OsPRR1 Alleles

#### 3.3.1. Two-day Transcription Patterns of Oscillator Genes in the Paddy Field

**Figure 2:**
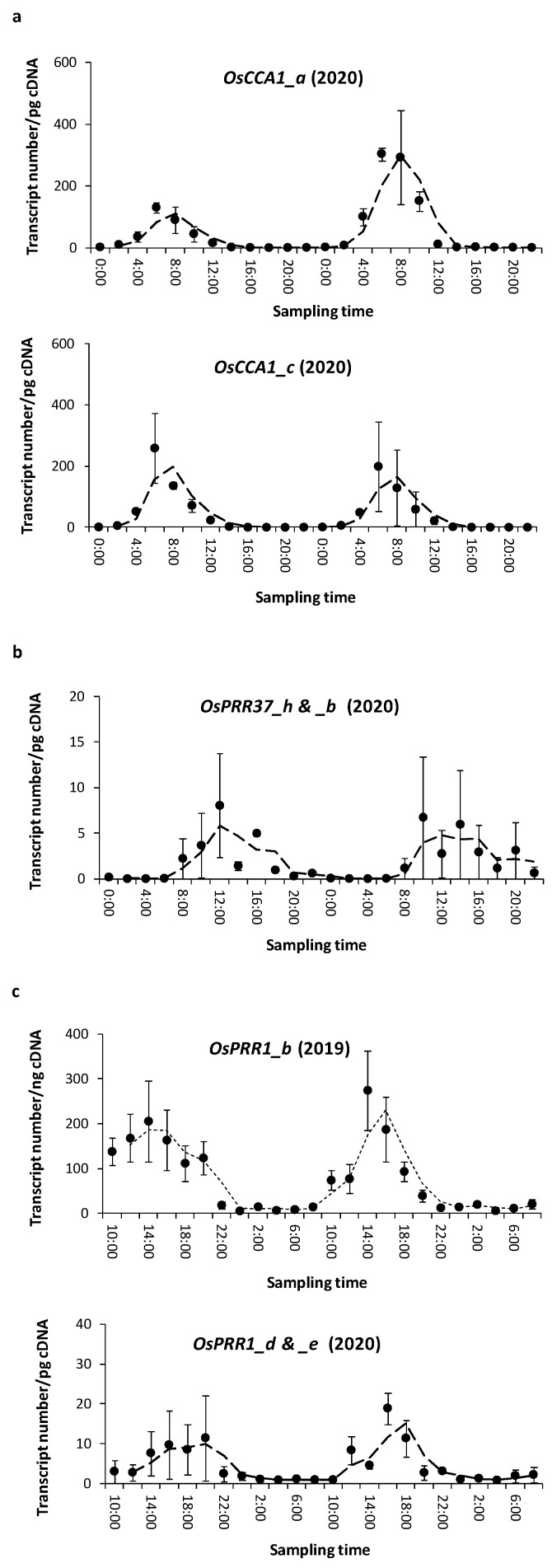
Natural expressions of alleles at three oscillator loci of *O*. *sativa* in the paddy field. (**a**) Expressions of *OsCCA1_a* and *OsCCA1_c* over 48 h. Leaf samples were taken every two hrs at a paddy field of IBCAS. An error bar is based on three biological replicates for *OsCCA1_a* and two biological replicates for *OsCCA1_c*. (**b**) Transcriptions of *OsPRR37_h* and *OsPRR37_b.* Leaf samples were taken as in (**a**) on two individuals carrying *OsPRR37_h* in D5 and *OsPRR37_b* in D3. The error bars are based on two alleles, showing variation of circadian expression. (**c**) Natural expressions of three alleles of *OsPRR1* sampled in two growth seasons. In 2019, four individuals carrying *OsPRR1_b* were sampled, with the error bars based on four biological replicates. In 2020, two individuals, one carrying *OsPRR1_d* and the other *OsPRR1 _e*, were sampled in D3, showing expression variation of the circadian expression, as in (**b**). Each data point here is based on at least two measurements. The dashed lines are the fitting of the moving averages at the periodicity of 2 in Excel.

To understand different modes of selection, we focused on three representative loci, *OsCCA1*, *OsPRR37*, and *OsPRR1*, for functional analysis. The in vivo expressions of some of the alleles identified above were examined at the paddy field. Transcript levels of two alleles of *OsCCA1* (*OsCCA1_a* and *OsCCA1_c*) were examined during the peak-growth days prior to heading (flowering) stage. The average transcript levels of *OsCCA1_a* in three individuals (one of D3 and two of D5) peaked around 6 a.m. (Figure 2a) and no difference was seen between the RIL lines. The overall level of *OsCCA1_a*’s transcription (50.7 ± 11.8 (s.e.) is higher than that of *OsCCA1_c*’s transcription (42.7 ± 12.2) but the variances were too large, due to periodicity, to permit a sensible comparison of the means. We later compared allelic expressions at a fixed time. The expression patterns above suggest that both alleles are functional in the field.

Since expression peaks of transcription were also similar for two alleles of *OsPRR37* (*OsPRR37*_*h* and *OsPRR37*_*b*) in 2020, which were around 12 p.m. with large experimental variances (Figure 2b), they were averaged to show the rhythmic expression pattern. For *OsPRR1*, allele *OsPRR1*_*b* was sampled in August 2019 in two RIL populations of different genetic backgrounds (line298 and line309), with two biological replicates per population. Their similar magnitudes of transcripts allowed pooling of data (Figure 2c), which show a transcript peak between 2 p.m. and 4 p.m. In August 2020, *OsPRR1*_*d* and *OsPRR1*_*e* were further examined, showing a largely consistent expression mode but with larger variances. The entire transcripts of the coding regions were further sequenced to ensure that allelic transcription was complete at each locus.

These natural expressions confirm regular transcriptions of these alleles, and the peak transcription times are comparable to their orthologs in *A*. *thaliana* in a growth chamber under a long-day condition [43].

#### 3.3.2. Allelic Expression Frequently Varies among Daily Environments and Genetic Backgrounds

**Figure 3:**
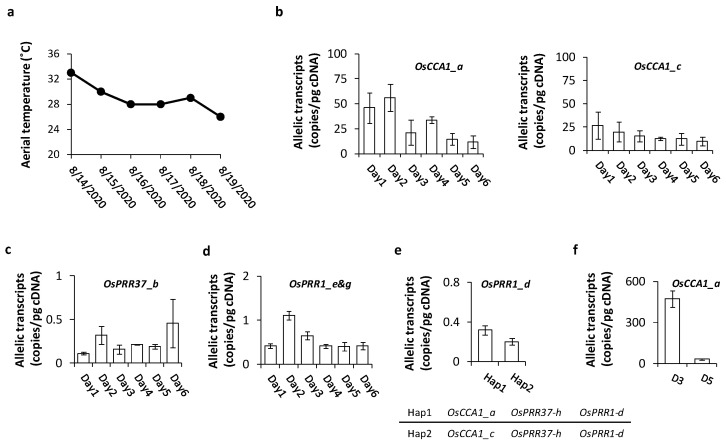
Effects of temperature and genetic background on allelic transcriptions in the paddy field. (**a**) Aerial temperatures of six sampling days. Daily high temperatures were from local meteorological records. (**b**) Quantifications of allelic expressions in the RIL population D5 over the six days. Two alleles (*OsCCA1*_*a* and _*c*) were sampled at 6 a.m. in six plants, each with three biological replicates, over the period shown in (**a**). Data are shown in mean ± se. (**c**) Transcript levels of *OsPRR37*_*b*. The samples were from the RIL population D3 at the time same as (**b**). (**d**) Transcript levels of *OsPRR1*_*e* and *OsPRR1*_*g.* The two alleles expressed at similar levels in the population D3 at the time same as (**b**) and were pooled here. (**e**) Effect of haplotype on transcription of *OsPRR1*-*d* in D5. Each haplotype (Hap) had two biological replicates sampled over six days at 6 a.m. from the same plants as (**b**). The bar of standard error represents 12 samples. The expression levels of *OsPRR1*_*d* differed significantly between Hap1 and Hap2 (one-sided *t*-test, *p* = 0.035). (**f**) Comparison of transcript levels of *OsCCA1*_*a* between two RIL populations. Sampled over six days shown in (**a**) in three biological replicates, *OsCCA1*_*a* was transcribed significantly highly in D3 population than in D5 population (*t*-test, *p* < 0.0001).

For more sensitive detections of allele-specific transcription of *OsCCA1*, we sampled three plants per allelic type in the RIL population D5, which had segregating *OsCCA1*_*a* and *OsCCA1*_*c*, at a fixed time (6 a.m.) around peak expression on six consecutive days in August 2020. Daily temperature was recorded at the sampling stage, along with extremes and the average of the day. The daily series allowed detections of influences of external factors on allelic expression. Only the highest aerial temperature, which showed a declining pattern over the period (Figure 3a), was correlated with the expression pattern of *OsCCA1*_c (Figure 3b), as indicated by a significantly positive Spearman rank correlation coefficient (r_s_ = 0.9, n = 6, *p* < 0.05). In the RIL population D3, daily variations of *OsPRR37*_*b* (Figure 3c) and *OsPRR1*_*e* and _*g* (Figure 3d) were observed, but their significant correlations with daily temperature were not detected.

Since the RIL population D5 was homozygous for *OsPRR1* (*OsPRR1*_*d*) and *OsPRR37* (*OsPRR37*_*h*) and the *OsPRR1*_*d* had been under positive selection shown above, we had a chance to check whether genetic backgrounds, represented by different haplotypes, can affect allelic expression. This effect is particularly relevant to oscillator loci due to their inter-relation. Two haplotypes, which were characterized by different *OsCCA1* alleles here, were compared for estimated transcript levels of *OsPRR1*_*d* (Figure 3e). The result indicates that *OsPRR1*_*d* expressed significantly more (57%) at the presence of *OsCCA1*_*a* than at the presence of *OsCCA1*_*e* (Figure 3e). Effect of genetic background on allelic expression is not limited to *OsPRR1*. In the RIL population D3, which segregated at *OsPRR1* (with *OsPRR1_e* and *_g*) but was homozygous for *OsCCA1* (*OsCCA1*_*a*) and *OsPRR37* (*OsPRR37*_*b*), its transcript level of *OsCCA1*_*a* differed significant from that of *OsCCA1*_*a* in D5 (Figure 3f).

#### 3.3.3. Mutations in 5′ Regions Can Alter Expression Levels of Alleles

**Figure 4:**
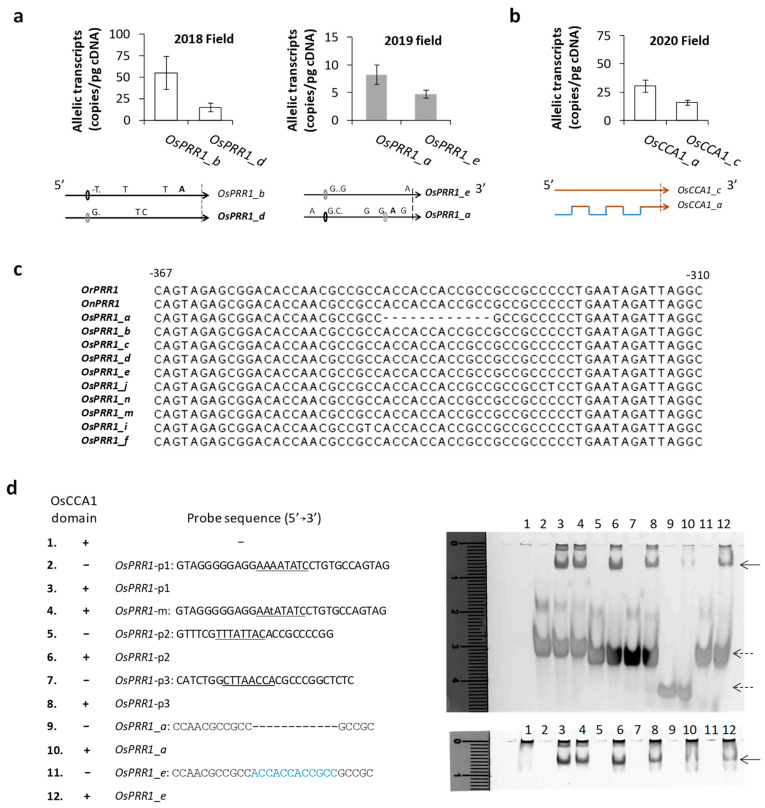
Effect of 5′ mutation on allelic expression. (**a**) Comparisons of allelic expressions of *OsPRR1* in evenings. The 2018 estimates were compared between *OsPRR1_b* in 36 biological samples (involving three accessions (two plants sampled per accession), each plant was sampled over the same six days (~6 p.m.–8 p.m., 4–9 August 2018)) and *OsPRR1_d* in 24 biological samples involving four accessions (one accession had two plants sampled and the other two accessions had one plant each). The 2019 estimates were compared between RIL lines carrying alleles *OsPRR1_a* or *OsPRR1_e.* Two plant replicates from each line were sampled at 6 p.m. over six days (6–11 August 2019). Each allele was measured in 12 biological samples. Error bar here is standard error of biological samples. The 5′ regions of the alleles in comparison are shown below each panel, following the format of Figure 1. (**b**) Comparison of allelic transcript levels of *OsCCA1* within the same RIL accession (D5). Three biological replicates were sampled at 6 a.m. for each allele. The error bars are for 18 biological samples taken on each replicate over six days (14–19 August 2020). The 5′ regions of the alleles are shown as in (**a**). (**c**) A deletion in the 5′ region of *OsPRR1*_*a*. The deletion is shown in dash in the alignment of the orthologous promoter regions of the parental species and *OsPRR1* alleles. The position of the last nucleotide is 310 bp before the beginning of start codon. (**d**) Probes tested for OsCCA1 binding domain in EMSA. The trials of binding reactions are numbered and shown in the right panel. The left panel shows the detail of the reactions: the domain added to the binding reaction is marked in “+”, one without the domain protein in “-”. The indel tested in probe 9 is shown in dashed line, and the canonical sequence (deleted in probe 9) is in blue in probe 11. Suspected *cis* elements (EE) for CCA1 are underlined in probes 1, 2, and 3 as positive controls. Binding results are shown in gels stained for DNA (upper) and protein (lower) in the right panel. A total of 12 binding reactions (1→12) were loaded to the gel for electrophoresis, with solid arrows indicating binding between the probe 11 (from *OsPRR1*_*e*) and the OsCCA1 domain and dashed arrows showing positions of free probes.

Due to the cyclic expression and sensitivity of oscillator genes to the natural environment, an evaluation of allelic transcription requires sampling at a fixed time (preferentially near the peak expression) of a day and several days in a row to be valid. Given substantial 5′ mutations of *OsPRR1* alleles, we examined allelic expression of *OsPRR1* in two field seasons. In August of 2018 at the same paddy field above, the mean transcript level of *OsPRR1*_*b* from four populations of line315 was compared against that of *OsPRR1*_*d* from three populations (one of line315 and two of line298). The results (Figure 4a) showed that transcript level of *OsPRR1*_*b* (55.1 ± 19.0 per pg cDNA, n = 24) was significantly higher than that of *OsPRR1*_*d* (14.7 ± 5.0, n = 36) according to one tailed *t*-test (*p* = 0.025). In August 2019 at the same paddy field, *OsPRR1_a* and *OsPRR1_e* were sampled in two populations (one of line298 and the other of line315). The mean transcript level (8.22 ± 1.76, n = 24) of *OsPRR1_a* was significantly higher than that (4.75 ± 0.89, n = 24) of *OsPRR1_e* (one-tailed *t*-test, *p* = 0.048). At *OsCCA1*, we had a chance to examine the effect of 5′ recombination on transcription, since *OsCCA1_a*, which has a recombined 5′ region, shows a significantly enhanced transcription (30.5 ± 5.5 (s.e.) per pg cDNA) than that (16.0 ± 2.5) of *OsCCA1_c*, which carries the parental 5′ region, within the population D5 at the same habitat above (Figure 4b, one-tailed *t*-test, *p* = 0.013, n = 18 for each allele).

To seek further evidence for associations of 5′ mutations with allele-specific expressions of oscillator gene, we examined a testable mutation at 5′ regions of *OsPRR1*. A specific deletion (12 bp) at the 5′ region of *OsPRR1*_*a* was found corresponding to a conserved sequence of ACCACCACCGCC in other alleles (Figure 4c). Because both parental sequences do not have this indel and it is also absent in other *Os* alleles, we considered it a new mutation that is specific to *OsPRR1*_*a*. Since the deletion contains partial binding sites (ACC) for MYB transcription factors, we suspect that it may interact with OsCCA1, a MYB-domain regulator. EMSA tests were subsequently carried out to compare binding capacities of the 5′ regions with or without the indel by the binding domain (R) of OsCCA1. Probes based on 5′ regions of *OsPRR1* alleles were prepared with or without the12-nt sequence (Figure 4d). Since evening-elements (EE) have been shown to be associated with CCA1 in *Arabidopsis* [73] and some 5′ regions of *OsPRR1* contain the EEs, we also made probes with the local EEs to serve as positive controls of the tests. Compared to the strong binding of the R-domain to probes with a canonical EE (OsPRR1-p1, -p2, and p3), the R-domain shows a weaker binding to the probe harboring ACCACCACCGCC and no binding to one with the deletion of the 12-nt at the 5′ region of *OsPRR1*_*a* (Figure 4d). Since CCA1 is a known MYB repressor of transcription of *TOC1* in *Arabidopsis* [74], reduced binding of OsCCA1 at the 5′ region of *OsPRR1*_*a* can enhance its *in vivo* transcription in comparison to that of *OsPRR1*_*e*, which has the same 5′ local region as all other *OsPRR1* alleles without the 12-nt deletion (Figure 4c).

With the experimentally supported link from 5′ mutations to allelic expressions above, we went to investigate whether changes in allelic expression can lead to changes of phenotypes detectable and desirable by humans.

### 3.4. Associations of Oscillator Alleles with Rice Heading Time and Yield in the Field

**Figure 5:**
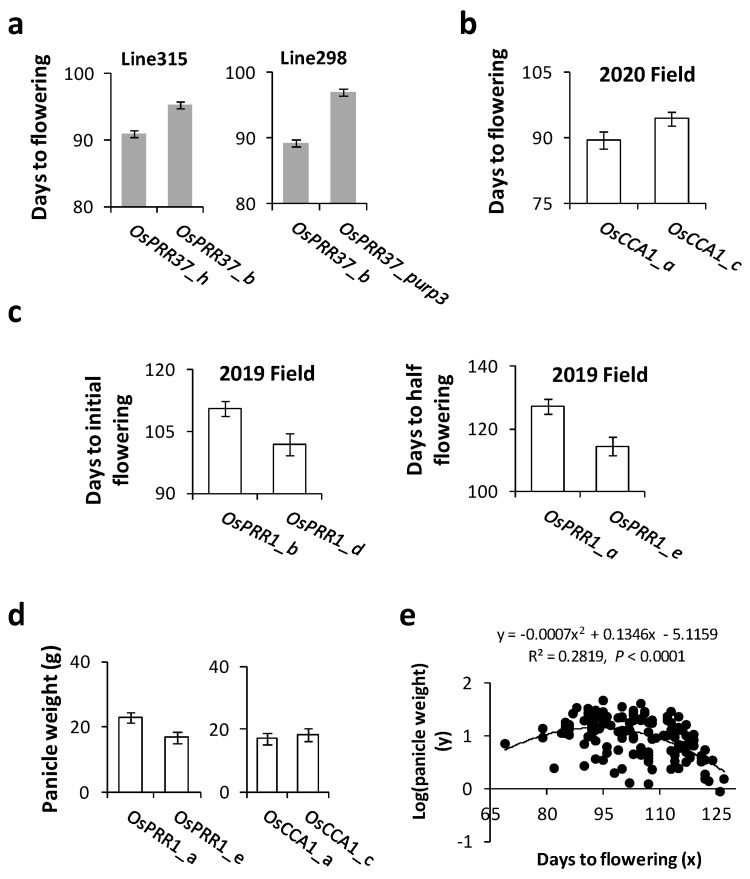
Allelic impacts on flowering days and panicle weight in Asian rice. (**a**) Days to the initial flowering of populations carrying different alleles of *OsPRR37* in 2017. For line298, three populations were included, one homozygous for *OsPRR37_b* (n = 148) and two for *OsPRR37_h* (n = 226). The difference is highly significant (*z*-test, *p* < 0.0001). For line315, one population homozygous for *OsPRR37_b* (n = 145) flowered significantly earlier than two populations of *OsPRR37_purp3* (n = 242) by *z*-test (*p* < 0.0001). Standard errors are shown in bars. (**b**) Comparisons of flowering days of plants carrying alternative *OsCCA1* alleles within the population D5. The standard error bar for *OsCCA1*_*a* had four biological replicates and that of *OsCCA1*_*c* had five. (**c**) Comparisons of impacts of *OsPRR1* alleles on days to flowering in 2019. The initial flowering days were compared between *OsPRR1_b* (10 populations: 7 of line298 and 3 of line309) and *OsPRR1_d* (13 populations: 12 of line315 and 1 of line298). For *OsPRR1_a* and *OsPRR1_e*, days to half-flowering (50% plants in flowering per population) were compared between three populations of line315 and ten populations of line298. (**d**) Comparisons of panicle weight between plants carrying alternative alleles of *OsCCA1* and *OsPRR1* in 2020. For *OsPRR1*, four plants of line 315 (homozygous for *OsPRR1*_*a*) had heavier panicles per plant than 16 plants (homozygous for *OsPRR1*_*e*) from five populations of line298. For *OsCCA1*, 16 plants of *OsCCA1*_*a* from four populations (2 of line315 and 2 of line298) were compared to 17 plants of *OsCCA1*_*c* from seven populations (4 of line315 and 3 of line298). (**e**) Detection of relationship between flowering days and panicle weight. A significant and non-linear relationship was shown in 141 plants sampled from 29 populations grown in the paddy field in 2020.

Since the most noticeable changes in rice to breeders are its flowering (heading) time and yield, we investigated impacts of combinations of alleles at different loci of the oscillator on these traits in the paddy field. During the 2017 growth season, flowering time was recorded at plant level for eight RIL populations (4 homozygous for *OsPRR37_h*, 2 homozygous for *OsPRR37_b*, and 2 homozygous for *OsPRR37_purp3*). The lines carrying *OsPRR37_purp3* took a significantly longer time (~97 ± 0.36 days) to reach the heading stage than lines carrying other alleles (Figure 5a). During the summer of 2020, plants homozygous for *OsCCA1_a* flowered significantly earlier than those homozygous for *OsCCA1_c* (Figure 5b, one-tailed *t*-test, *p* = 0.043). In 2019, lines homozygous for *OsPRR1_b* reached the initial heading stage at significantly later dates (~111 days) than those of *OsPRR1_d* (~102 days) according to one-tailed *t*-test (*p* = 0.007); at the same site, lines homozygous for *OsPRR1_a* reached the initial heading stage at significantly later dates (~127 days) than those of *OsPRR1_e* (~114 days) according to one-tailed *t*-test (*p* = 0.01) (Figure 5c). These patterns suggest significant impacts of the alleles at the three oscillator loci on heading time of Asian rice.

Associations of oscillator alleles with yield were investigated in the growth season of 2020 for *OsCCA1* and *OsPRR1*. The panicles collected from sampled plants, with their flowering time reported above, were found heavier for later blooming plants (Figure 5d) for both loci. While the impact was relatively small between *OsCCA1_a* and *OsCCA1_c*, plants homozygous for *OsPRR1_a* produced significantly more grains than ones for *OsPRR1_e* (one sided *t*-test, *p* = 0.003, n_1_ = 4, n_2_ = 16). To detect a possible connection between plants’ heading date and panicle weight regardless of genotypes at the oscillator loci, we collected these data from 21 RIL populations and 8 varieties/landraces in 2020 and detected a significantly non-linear relationship between flowering time and panicle weight at individual level (Figure 5e). The relationship suggests that the best theoretical yield can be achieved in the Beijing area with heading days of around 95 to 96 (95% confidence interval ranging from 86 to 103 days) counting after grain germination. Clearly, because of their impacts on both flowering and yield, oscillator genes would be unknowingly targeted by human selection, which can in turn influence the pace by which an oscillator system evolves. Meanwhile, natural selection may also take place when range expansion of a species occurs. To separate the effect of natural selection from that of human selection, more analysis was conducted below.

### 3.5. Divergent Patterns of Oscillator Genes between Oryza and Arabidopsis

Since *A*. *thaliana* is an as widely distributed annual [75] as Asian rice but has experienced little human impact, we examined its oscillator, assuming similar roles of oscillators between species. As the sample size of *Os* genomes analyzed above, genomes of ten ecotypes of *A*. *thaliana* (Table S9) were surveyed for the five oscillator orthologs. Recent *At* mutations were tentatively identified under the assumptions that recent mutations are at relatively low frequencies (<30%) in *A*. *thaliana* when ancient low polymorphisms can be excluded. The exclusion was judged by homologous sequences of *A*. *arenosa*, a species belonging to the same genus of *A*. *thaliana*. A total of 67 tentative mutations can be aligned unambiguously at 5′ or coding regions of four loci (alignments of *PRR5* were ambiguous thus not included) between the species, 61 of which are supported by the homologous sites of *A*. *arenosa* and 6 which are considered ancestral and excluded (Tables S10–S13), which led to an error rate of 0.09. The six errors in mutation assignment were corrected in the subsequent analysis of genetic diversity.

In *A*. *thaliana*, the oscillator loci appear to vary comparably at 5′ or coding regions in rate of accumulating recent mutations (<threefold), and mutation density at 5′ regions is generally higher than that of coding regions (Table 3). By contrast, oscillator loci of *O*. *sativa* differ greatly at 5′ regions, with 5′ of *OsCCA1* holding the least number of mutations and 5′ of *OsPRR1* having a significantly higher number of mutations, causing more than twenty-fold variation in mutation density (Table 1). Meanwhile, *OsPRR37* has a 5′mutation density even lower than that of its coding (Table 1 and Table S4, Figure 1h). To make sure that the between-species comparisons are valid in function, we further evaluated divergence of *CCA1* and *PRR1* between species below by experiments of compensation and related tests.

### 3.6. Compatibilities of OsCCA1 and OsPRR1 to Their Orthologs in A. thaliana

#### 3.6.1. Effects of *OsCCA1* alleles in *A. thaliana*

**Figure 6:**
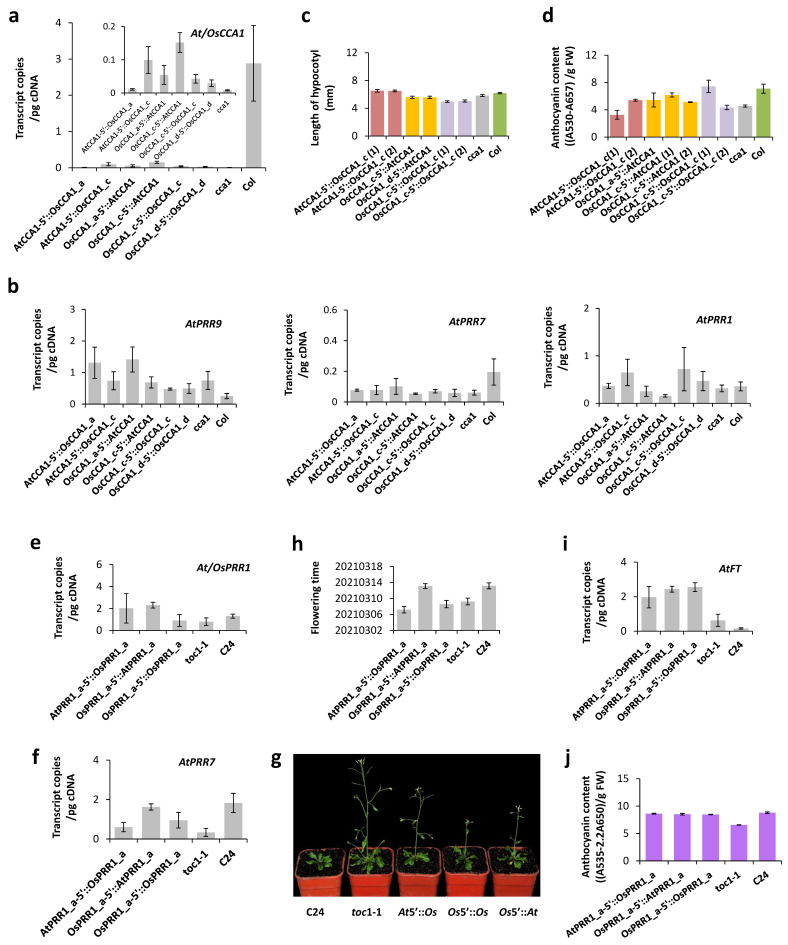
Partial complementation of *Os* alleles in *cca1* and *toc1-1* of *A. thaliana*. (**a**) Expressions of *OsCCA1* in fresh leaves among T1 transformants. Transcript levels of *CCA1* were estimated at 7 a.m. in mature plants of six types of transformed *cca1* of *A*. *thaliana*, with *cca1* and the wild-type (Col) as negative and positive controls, respectively. The standard error bar per type includes at least three biological replicates around the mean transcript number of *OsCCA1* or *AtCCA1* (depending on the coding regions introduced). (**b**) Expressions of other *Os* loci. The samples are the same as in (**a**). (**c**) Length of hypocotyl of day-5 seedlings. Each transformant type had at least 15 plants surveyed. Duplicated transformations are numbered in parentheses. (**d**) Anthocyanin content of day-3 seedlings. Each measurement contained at least 15 seedlings. Three measurements were taken for each transformant type. Duplicated transformations are numbered in parentheses. (**e**) Expressions of *OsPRR1* in fresh leaves among T3 transformants. The samples were taken at 4 p.m. and processed as in (**a**). (**f**) Expressions of *AtPRR7* in transformed *toc1*-*1* plants. The samples are the same (**e**)*. (***g**) Growth conditions of transformed *toc1*-*1* (T2) on day-35 in a growth chamber. *At*5′ is for the 5′ region of *AtCCA1*_*a* and *At* is for the coding regions of *AtCCA1*_*a*. So is *Os*5′ or *Os* for *OsCCA1*_*a*. (**h**) Time to flowering. The germination started on 30 January 2021. The growth condition is the same as above under 23 °C 16 h light/21 °C 8 h dark. The means are based on a sample size of 13–20 plants, with standard errors shown. (**i**) Expressions of *AtFT* in transformed *toc1*-*1* plants. The samples were taken as in (**e**) at 7 a.m. (**j**) Anthocyanin contents in young leaves of T2 transformants. The anthocyanin content was measured as absorbance under 535 nm light and corrected by A650.

5′ regions (~1 Kb) and coding sequences of *OsCCA1_a* and *OsCCA1_c* were introduced independently, along with the whole allele of *OsCCA1_c* (introns excised), into the *cca1-1* mutant (CS67781) of *A*. *thaliana* with genetic background of the ecotype Columbia. After transformants were gathered and confirmed, we examined in T1 before flowering of plants (32 days after germination) expression levels at 7 a.m. of *OsCCA1* driven by the 5′ region of *AtCCA1* or those of *AtCCA1* driven by a 5′ region of *OsCCA1.* Transcript levels of the transformants were all lower than the level of the wild type (Figure 6a). Nonetheless, plants of *OsCCA1_c* 5′::*AtCCA1* can generate significantly higher transcript level of *AtCCA1* than *cca1-1* (one-tailed *t*-test, *p* = 0.039), suggesting some functionality of *OsCCA1_c* in *Arabidopsis*. This weak function of *OsCCA1_c* did not lead to ostensible changes in expressions of *AtPRR9*, *AtPRR7*, and *AtPRR1* in *Arabidopsis* (Figure 6b). The coding region of *OsCCA1_a* was hardly expressed when driving by the 5′ region of *AtCCA1* and its 5′ region showed only a negligible effect in driving expression of the *CCA1* coding sequence of *Arabidopsis*. The whole allele of *OsCCA1*_*d* also failed to show its function in *Arabidopsis.* These patterns suggest that only parental allele *OsCCA1_c* is more or less recognized by *A*. *thaliana*.

Phenotypically, *cca1-1* has a shorter hypocotyl than that of the wild-type Columbia (Col). The hypocotyls of most transformants failed to elongate except plants of *AtCCA1* 5′::*OsCCA1*_*c*, which developed over-elongated hypocotyls that surpassed these of the wild type (Figure 6c). The overcompensation suggests a less than appropriate interaction of *OsCCA1*_*c* with local genes of *A*. *thaliana*. For transient accumulation of anthocyanins in T2 seedlings, plants of *OsCCA1*_*c* 5′::*AtCCA1* as well as the entire allele of *OsCCA1_c* can significantly restored the phenotype of pigment accumulation in transformed *cca1-1* (Figure 6d). These results again support partial functions of *OsCCA1*_*c* in *Arabidopsis*, which is the allele from *O. rufipogon* that has not been changed by human selection.

#### 3.6.2. Effects of *OsPRR1_a* in *A. thaliana*

The 5′ region and the coding sequences of *OsPRR1_a* were introduced into the *toc1-1* mutant (CS3756) of *A*. *thaliana* separately, along with the entire allele (introns excised), at the genetic background of the ecotype C24 to allow evaluations of possible effects. The expression of *AtPRR1* or *OsPRR1* was assessed by the transcript level at 4 p.m., showing that the 5′ region of *OsPRR1_a* can drive the expression of *AtPRR1* beyond level of the wild type (Figure 6e). Meanwhile, transcription of *AtPRR7* was more or less restored in the three types of transformants, compared to the wild-type level (Figure 6f). These patterns support partial functionality of *OsPRR1*_*a* in *A*. *thaliana*.

Since *toc1-1* is known to have an earlier flowering phenotype under long-day condition [51], we compared phenotypes of flowering date among the transformants, the mutant, and the wild-type C24 (Figure 6g). Compared to C24, the transformants more or less flowered earlier (Figure 6h), and quantifications of transcript levels of *FT* (Figure 6i), the flowering-promoting gene, support the genetic basis of the flowering phenotype. The pigment accumulation was also measured at 17-day-old seedlings following a cold exposure, which suggests a significant restoration of anthocyanin accumulation in the transformed *toc1-1* (Figure 6j).

### 3.7. Linking Allelic Diversity of Oscillator Loci to Geographic Expansion of Rice Cultivation

#### 3.7.1. Geographic Distributions of Oscillator Alleles at *OsPRR1* and *OsPRR37* across Main Regions of Rice Cultivation

**Figure 7:**
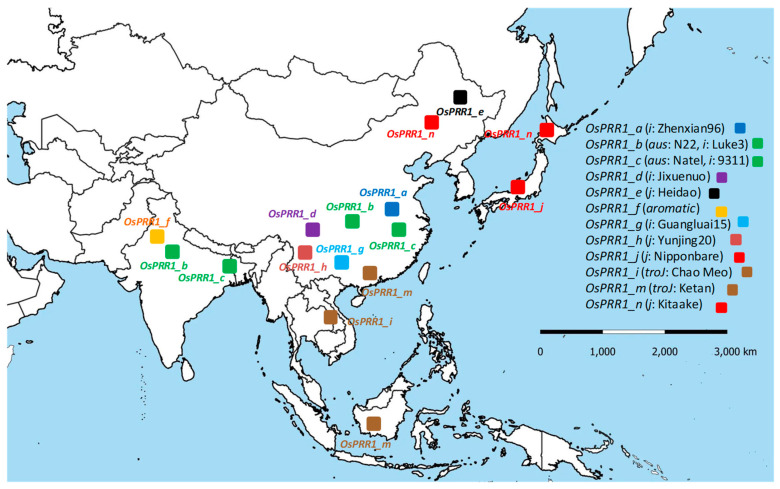
An approximate geographic distribution of 12 *OsPRR1* alleles. The sequences of the alleles see Tables S6 and S8. The subgroup and the allelic carrier are shown in the parentheses, with *i* for *indica* (*sensu stricto*), *j* for *japonica* (*sensu stricto*), *troJ* for *tropical japonica*, and a representative variety following the comma.

Although all oscillator loci of *A*. *thaliana* and *O*. *sativa* have been under natural selection, significantly altered allelic mutation rates at 5′ regions of *OsPRR1* and *OsPRR37* (Table 2) in comparison to the orthologs in *A*. *thaliana* indicate that allelic expressions have been targeted during domestication of rice because no such signals of selection can be detected in *A*. *thaliana*. If human selection acted on *OsPRR1* in Asian rice to aid its range expansion, we predicted that more alleles of *OsPRR1* were to be found in southern parts of the distribution range of Asian rice, since bigger variations in climate and latitude are in regions south of Yangtze River. This hypothesis is based on impacts of *OsPRR1* on flowering and yield documented here as well as positive selection detected on the gene. By mapping 12 identified *OsPRR1* alleles according to their approximate geographic distributions shown by the carriers of landraces and varieties, we found that the prediction largely holds (Figure 7). As a comparison, 10 alleles of *OsPRR37* were also mapped (Figure S3), some of which overlap with alleles (*PRR37-2a* of H143, *PRR37-1* of Miyang23) in an early report [70]. Although there is some resemblance between the two distributions due to shared data of the ten genomes of *O*. *sativa*, more alleles (7) of *OsPRR1* were seen in southern parts of China than those (3) of *OsPRR37*.

#### 3.7.2. A Better Fitting of Allelic Distribution at *OsPRR1* Than at *OsPRR37* to Main Regions of Rice Cultivation

When locally adapted alleles assist range expansion via 5′ or coding mutations, distribution of the relevant alleles is expected to be more or less restricted geographically. Due to strong purifying selection at the 5′ regions of *OsPRR37*, alleles *OsPRR37*_*a*, _*h*, and _*f* all carry the same 5′ region as that of *O*. *rufipogon*. Nonetheless, varieties bearing these alleles, e.g., Nipponbare, Kitaake, and Ketan, are distributed over a wide range of latitudes (Figure S3). The distribution pattern suggests little impact of their 5′ regions to range expansion of rice. Likewise, alleles (*OsPRR37*_*b*, _*c*, _*d*, _*e,* and _*i*) having *On*-based 5′ regions of *OsPRR37* also distribute widely with their carriers. These two patterns argue against the likelihood that the expression variation of *OsPRR37* assisted range expansion of *O*. *sativa*. For the coding regions, three alleles (*OsPRR37*_*c*, _*d*, and _*i*), each of which accommodated an indel at different sites of coding regions that disrupted the protein (Figure 1), were distributed over broad latitudes along with the carriers (e.g., from temperate *japonica* to *tropical japonica*). This pattern also conflicts with a significant role of *OsPRR37* in the range expansion. By comparison, *OsPRR1* alleles can express variably via 5′ mutations and have no indels in the coding regions in the materials examined here, and their allele-specific mutations (Figure 1, Table S8) can provide qualitative and quantitative adjustments needed for local adaptation, consequently facilitating range expansion. Among five oscillator loci examined here, the strong positive selection and the geographic distribution of *OsPRR1* alleles support a significant role of *OsPRR1* in range expansion of Asian rice.

## 4. Discussion

### 4.1. Human Selection as a Major Force for Evolution of Oscillator in a Crop

Since a circadian clock affects a significant proportion of genomes, about 43% protein-coding genes in mouse [76] and about one third of expressed genes in *A*. *thaliana* [77], it can hardly not be touched during domestication by human selection or/and natural selection. Here, we show how human selection may influence the oscillator in Asian rice. Through quantifying mutation density in the sample size same as that of Asian rice on oscillator loci of *A*. *thaliana* over a comparable range of geographic distribution (Table S9; Figure 7), the natural distribution of mutations across loci becomes evident and may serve as reference for identifying selection in Asian rice. Compared to a much less variable mutation density across *Arabidopsis* loci (Table 3), the unbalanced mutation densities observed in *O*. *sativa* at loci of *CCA1/LHY*, *PRR37*, and *PRR1* (Table 1) can be ascribed to human selection, as these features are entirely absent in *A*. *thaliana,* although the two species have experienced comparable ranges of latitude (day length) and temperature, the most influential environmental factors for oscillators. They also share similar life histories (annual, selfing, and multi-seeded) but have different species histories. Considering the natural history of *A*. *thaliana*, estimated to be about 600 Kyr [78] whereas that of *O*. *sativa* is about 8–10 kyr [79], the similar levels of mutation densities observed in the two species (Table 1 and Table 3) suggest that the intensity of human selection on oscillator of Asian rice was nearly 60 times higher than that of natural selection on oscillator of *Arabidopsis*.

The strong positive selection at 5′ regions of *OsPRR1* supports the assessment above, which led to a range of allelic expressions. Because natural dispersal of *O*. *sativa* is very limited, its range expansion was mainly enabled by human-assisted cultivation. The increasing allelic diversity at *OsPRR1* was most likely encouraged by early breeders, since not only flowering time but also yield can be associated directly with certain alleles of *OsPRR1* (Figure 4 and Figure 5). The capacity of rice’s fine adjustments to local climates clearly depends on collective outputs of oscillator genes, as *OsCCA1* may respond to temperature (shown here) and modify flowering time [37] and OsPRRs may interact with genes on the flowering pathway [32,80,81]. Among the five oscillator genes, both *OsCCA1* [37] and *OsPRR37* [70] have been proposed to be responsible for range expansion of *O*. *sativa*, but neither their allelic distributions nor selection patterns are as consistent as those of *PRR1* in interpreting range expansion of Asian rice. Noticeably, *OsCCA1* and *OsPRR37* still engage some parental alleles, whereas alleles documented at *OsPRR1* so far have accumulated *Os*-specific mutations, deviating from parental alleles. Given documented response of *toc1*-*1* to low sensing of day length in flowering at *AtPRR1* [51], human selection on a specific *OsPRR1* allele may assist fine-toning downstream genes to deliver an appropriate flowering time for local cultivation of Asian rice.

Besides summed mutations, temporal distributions of mutations across the oscillator loci can be assessed with gene-genealogies and allow an inference on temporal changes of human. Since mutations shared among subgroups occurred earlier than those specific to one subgroup, the degree of shared mutations is indicative of the approximate period when selection for specific mutation occurred. About half of the mutations at loci of *OsPRR95*, *OsPRR37*, and *OsPRR59* are shared among subgroups, which indicate relatively early selection at the loci. In comparison, mutations of *OsCCA1* and *OsPRR1* (to a less extent due to one early mutation) are shared less among alleles, implying a later differentiation of the alleles at the loci. This later period appears overlapping with the range expansions of the crop [60].

### 4.2. Regulations of Circadian Clock in Quantity and Quality via Allelic Series

Because both positive and negative selections were detected at 5′ regions of oscillator loci, changes at the 5′ region become particularly noteworthy. We showed here significant impacts of *OsPRR1*_*a* vs. *OsPRR1*_*e* on flowering days and panicle weight; the two alleles encode the same protein and differ only at their 5′ regions (Figure S1); more 5′ mutations accumulated at *OsPRR1*_*a* evidently caused its higher level of transcription and ultimately changes in phenotypes. Quantitative changes in transcription of the oscillator may effectively fine-tune downstream targets of the circadian clock without causing dramatic changes in protein-protein or protein-DNA interactions. The presences of parental alleles at the locus are likely not incidental in Asian rice since parental alleles could help early assembly of the circadian clock in the incipient crop by reconnecting the much-shifted clock to their targets quickly. For instance, OsLHY (CCA1) interacts with the promoter of *OsGI* to influence rice’s heading date under different lengths of day [82], maintaining a parental allele in hybrid progeny that could help maintain this connection.

A significant response of allelic expression to environmental variation was observed here for *OsCCA1*_*c* allele over the six-day interlude, which is likely not restricted to the allele/locus. In potato tuber, a tuber-promoting gene (*StSP6A*) can be suppressed by temperature-sensitive expression of *StTOC1* (*PRR1*) to reduce tuber yield [83]. A longer sampling period for natural expression variation is likely required for detection of additional responses of allelic expressions across loci.

In contrast to expression of an allele, changes in the coding regions, particularly at interacting domains, may cause more severe consequences in protein-protein or protein-DNA interactions. At *OsPRR37*, *japonica* alleles (*OsPRR37*_*a*, *OsPRR37*_*h*, *OsPRR37*_*f*) maintain the ancestral 5′ region (same as that of *O*. *rufipogon*) but kept four amino-acid-changing mutations to cope with new environments, which suggest that new interactions rather than expression levels were encouraged by past breeders for the locus. Specific examinations of impacts of *Os* mutations on the signaling pathways can be carried out to pursue the interactions.

### 4.3. Birth of a New Allele Can Initiate at Either 5′ or the Coding Regions

Identifications of oscillator alleles in Asian rice provide living examples of evolution of new alleles from old ones. For hybrid progeny, recombinants are naturally the initial source for new alleles. At *OsCCA1*, three historical episodes of recombination occurred prior to *aromatic* rice, giving rise to alleles of *OsCCA1*_*a*, *OsCCA1*_*d*, and *OsCCA1*_*f*, along with new mutations. We have shown that recombined 5′ region of *OsCCA1*_*a* is associated with a higher transcription of the allele than that of the parental allele *OsCCA1*_*c* (Figure 4).

Our analysis indicates that allelic differentiation can start at either coding regions, as shown at *OsPRR37*, or in 5′ regions, as exhibited by *OsPRR59* or *OsPRR95* here. Two *japonica* alleles, *OsPRR37*_*a* and *OsPRR37*_*h,* clearly came from the same recombination episode that gave rise to an early allele, as shown by the shared events of recombination and a nonsynonymous mutation (Figure 1). Their differentiation from each other was marked by three nonsynonymous mutations (one occurred to *OsPRR37*_*a* and two to *OsPRR37*_*h*), possibly promoted by positive selection on the coding of *OsPRR37*_*h*. Similarly, four alleles (*OsPRR37_b*, _*c*, *_d*, _*i*) came from another episode of recombination and the early allele is represented by *OsPRR37*_*b*.

Two alleles of *OsPRR95* further demonstrate initial allelic divergence at a 5′ region. The alleles, *OsPRR95*_*a* and *OsPRR95*_*b*, encode the same protein but differ largely at their 5′ regions. The same pattern can be found at locus *OsPRR59*, between *OsPRR59_a* and *OsPRR59_b*. Clearly, selection for 5′ divergence targets transcription of an allele, whereas selection for coding mutations likely influences the downstream genes that interact with the oscillator locus. The parallel cases above illustrate how 5′ and coding regions can evolve independently from each other under different selection.

### 4.4. Gene-Phenotype Associations

Because flowering pathways differ to some degree between species [84], gene-phenotype associations may vary as well with species. It is important to validate similar or dissimilar associations between species. In *Arabidopsis*, mutant *toc1-1* has a reduced response to day length in flowering time [51] and so blooms earlier. If lack of TOC1, an ortholog of OsPRR1, can lessen responses of flowering to the day length in *Arabidopsis*, reduced expression of *OsPRR1* might be expected to have a similar effect on flowering. This anticipated effect appears held in Asian rice, since carriers of less expressed *OsPRR1*_*e* flowered earlier. This association can well interpret human selection on *OsPRR1* during range expansion.

In *Arabidopsis*, GIGANTEA (GI) promotes flowering by activating *FT* [85], a florigen that promotes the transition of an apical meristem to a floral bud, and PRRs (PRR1, 5, 7, and 9) may collectively promote transcription of *FT*, by interacting with CO [86], a repressor of *FT*’s expression. In rice, however, GI suppresses flowering under long-day condition by boosting expression of *CO* and repressing transcription of *FT*, opposite to the role of GI in *Arabidopsis* [54]. Nonetheless, higher expression of *OsPRR37* was reported to be associated with later flowering under long-day condition [37]. Here, we observed a significantly lower transcript level of *PRR7* in *toc1-1* than in the wild type, and the *FT* level was in the opposite pattern, causing early flowering of *toc1-1* under long-day condition in *Arabidopsis* (Figure 6f–i). These patterns imply the same relationship of *PRR7*’s expression with flowering time between species. It is further shown here that alleles of both *OsCCA1* and *OsPRR1* may influence flowering time but their effects on panicle weights differ.

The greatest difficulty with circadian clock analysis is to tell apart the direct and correlated changes, when both can lead to a similar phenotype. For instance, low expressions of *OsCCA1* may result in elevated OsPRR95 (Figure 6b) and OsPRR37 [37] under long day; it is uncertain which one is the direct cause for reduced anthocyanin content in seedlings without further experimentation. For influence of *OsCCA1* alleles on yield, we did not observe association of their transcript levels with panicle weights, and neither did Lee et al. [37] when they compared plants of *oscca1* to those of over-expressed *OsCCA1*. Meanwhile, alleles of *OsPRR1* show significant effects on panicle weights (Figure 5d).

### 4.5. Implications for Future Breeding

Besides allelic effects of *OsPRR37* on flowering, allelic comparisons of *OsPRR1* and *OsCCA1* here suggest that lower expression of *OsPRR1* and higher expression of *OsCCA1* tend to shorten days to heading in Asian rice, and *OsPRR1*_*e* and *OsCCA1*_*a* can be candidate alleles for breeding rice varieties of a shorter growth season. The positive association of *OsPRR1*′s transcription level with flowering time is likely not specific to *OsPRR1*_*e*, since *OsPRR1*_*b* and *OsPRR1*_*d* displayed the same pattern (Figure 5). These results echoed with previous tests under an artificial treatment. For instance, experimentally over-expressing *OsCCA1* can lead to early flowering while its abortion can delay the heading by reducing transcriptions of *Hd3a*, a homolog of *Arabidopsis FT* [87], and *Ehd1* (Early heading day 1), a flowering-promoting gene under short-day condition [88], but boosting transcription of *OsPRR37* under a long-day condition [37].

Though allelic expression unknown, *OsPRR37_purp3* is associated with later blooming while *OsPRR37_h* (H143 allele) appears to promote early flowering (an effect previously documented [89]). Here, we show that *OsPRR37_h* expresses at a relatively low level over a 48 h period (Figure 2). These results predict that *OsPRR37*_*purp3* may express at a higher level than *OsPRR37_h* does. Though alleles of *OsPRR95* and *OsPRR59* are not tested here, a knockout mutant *osprr59* has an early flowering date [32]. They are expected to influence flowering time together with other three loci. As an optimal yield or combination of traits ultimately depends on the collective output of a circadian clock, a more comprehensive understanding of allelic effects of oscillator loci will enable a molecular design of a rice variety of not only an appropriate growth period but also desirable traits.

Here, we show the field evidence for the anticipated relationship between heading time and yield, obtaining the desired local flowering time for the highest weight of panicle (Figure 5e). The method paves the way of choosing appropriate alleles in a breeding design. Other crops such as maize and sorghum can benefit from the methods and results presented here, as the principle of oscillator-flowering time/yield relationship may hold for them, too. In foreseeable future, the alleles of oscillator genes can benefit agriculture via promoting better food production that ultimately requires less land while being more environmentally friendly.

## 5. Conclusions

Emergence of a novel allele may start via mutation(s) at either 5′ or coding region or via recombination. Multiple alleles have existed at the oscillator loci, potentially providing variation as well as plasticity to the function of a circadian clock. Evolution of a new circadian clock in an early crop can be directed by both speciation events and selection (particularly human-initiated) via emerging alleles. Compared to oscillator genes in *A*. *thaliana*, which evolved more coordinately in the natural environment but at a much slower pace, the orthologs in *O*. *sativa* evolved much faster due to strong human selection, which promoted fast 5′ evolution at one locus but slow 5′ evolution at another without breaking down the system. In both *O*. *sativa* and *A*. *thaliana*, *PRR1* accommodates more variation than other loci, which may associate with its recognized role here in range expansion.

For gene-phenotype relationships, we have shown that natural changes (including mutations at 5′ regions and natural temperature) can cause varied transcription levels of oscillator alleles (e.g., *OsCCA1*_*c* and *OsPRR1*_*a*), which may influence flowering time in ways predictable from field tests. Some of the allelic effects (e.g., *OsPRR1*_*a* and _*e*) can alter the final yield via the flowering time–panicle weight relationship. Through a chain of the documented events, allelic composition at oscillator loci can be linked to plant phenotypes such as days to flowering and total panicle weight per plant. We have demonstrated that allelic variation is essential to phenotypic variation.

## Figures and Tables

**Table 1:** Allelic diversity of the oscillator genes across ten genomes of *O. sativa*.

Locus	Number of Alleles	5′ Region			Coding Region(s)		
		Length * (nt)	Mutations	Mutation Density	Length * (nt)	Mutations	Mutation Density
*OsCCA1*	6	1014	1	0.0010	2160	7	0.0032
*OsPRR95*	8	1031	16	0.0155	1872	4	0.0021
*OsPRR37*	9	1036	4	0.0039	2230	15	0.0067
*OsPRR59*	6	1015	15	0.0148	2088	9	0.0043
*OsPRR1*	7	1305	28	0.0215	1557	8	0.0051

Note: * length is based on that of the alignment of allelic regions per locus.

**Table 2:** Statistical tests on selection at five loci of the *Os* oscillator.

	Mutation Rate Per Allele			Probability for One Tailed *t*-Test		
Locus	5′	Coding	Intergenic ^a^	5′ vs. Int. ^b^	Coding vs. Int.	5′ vs. Coding
*CCA1/LHY*	0.00026 (0.00016)	0.00046 (0.00023)	0.00109 (0.00045)	0.036	0.059	0.086
*PRR95*	0.00194 (0.00066)	0.00027 (0.00013)	0.00108 (0.00035)	0.141	0.087	0.034
*PRR37*	0.00043 (0.00013)	0.00075 (0.00022)	0.00216 (0.00027)	<0.001 ^c^	0.163	0.018
*PRR59*	0.00246 (0.00058)	0.00074 (0.00030)	0.00211 (0.00054)	0.332	0.224	0.131
*PRR1*	0.00307 (0.00029)	0.00077 (0.00020)	0.00230 (0.00067)	0.009 ^c^	0.115	<0.0001 ^c^

Note: ^a^ The baseline for *CCA1* is from the third intron of each allele, the alignment of which is 3963 bp in length. ^b^ Intergenic (Int.) ^c^ The test is significant after a correction for multiple comparisons.

**Table 3:** Allelic diversity of the oscillator genes across ten genomes of *A. thaliana*.

Locus	Number of Alleles	5′ Region			Coding Region(s)		
		Length ^a^ (nt)	Mutations ^b^	Mutation Density ^c^	Length ^a^ (nt)	Mutations ^b^	Mutation Density
*CCA1*	8	1002	11	0.0130	1827	4	0.0022
*PRR9*	7	1012	5	0.0049	1407	8	0.0057
*PRR7*	9	1018	10	0.0098	2184	13	0.0060
*PRR5*	8	1011	10	0.0099	2010	13	0.0065 ^d^
*PRR1*	10	1020	12	0.0118	1857	4	0.0022

Note: ^a^ length is based on that of the alignment of allelic regions per locus. ^b^ Numbers of mutations of 5′ regions of *AtCCA1* and *AtPRR9* have been corrected after error checking with genome of *A*. *arenosa*; so were coding regions of *AtPRR7* and *AtPRR9.* ^c^ Mutation density is based on 10 sequences per genic region of estimated mutations that are less than 0.3 in frequency. Its unit is the number of mutations per site per 10 sequences per comparison period. The period here refers to the longest generations required to reach 0.3 in frequency. ^d^ Because of two non-functional alleles in Ler and Tanz, mutation rates are higher.

## Data Availability

All data generated or analyzed during this study are included in this publication.

## Supplementary Materials

The following supporting information can be downloaded at: https://www.mdpi.com/article/10.3390/genes14112027/s1, Figure S1: Additional alleles identified in independent surveys of Asian rice. Figure S2: Identifications of background mutations in the genomic regions taken as references of alleles at an oscillator locus. Figure S3: Approximate distribution of *OsPRR37* alleles surveyed in this study. Table S1: Genomes analyzed in this study. Table S2: Polymorphic sites at *OsCCA1* (Os08g06110.2) from the ten genomes. Table S3: Polymorphic sites at *OsPRR95* (Os09g36220.1) from the ten genomes. Table S4: Polymorphic sites at *OsPRR37* (Os07g49460.1) from the ten genomes. Table S5: Polymorphic sites at *OsPRR59* (Os11g05930.1) from the ten genomes. Table S6: Polymorphic sites at *OsPRR1* (Os02g40510.1) from the ten genomes. Table S7: Additional alleles of *OsPRR1* detected in this study. Table S8: Ten genomes of *A. thaliana* surveyed for alleles of oscillator genes in this study. Table S9: Additional accessions of *O*. *sativa* surveyed in this study. Table S10: Mutations identified at *AtCCA1* and checked against genome o*f A. arenosa* (*Aa*). Table S11: Mutations identified at *AtPRR9* and checked against genome o*f A. arenosa* (*Aa*). Table S12: Mutations identified at *AtPRR7* and checked against genome o*f A. arenosa* (*Aa*). Table S13: Mutations identified at *AtPRR1* and checked against genome o*f A. arenosa* (*Aa*).
