# Supplementary material for: Linking New Alleles at the Oscillator Loci to Flowering and Expansion of Asian Rice
Source: Genes (Basel). 2023 Oct 31;14(11):2027. doi: 10.3390/genes14112027 (PMC10671530; doi:10.3390/genes14112027)
Supplement: Supplementary file 1 [file genes-14-02027-s001.zip › genes-2630585-supplementary.pdf]

Supplementary Information to

Linking new alleles at the oscillator loci to flowering and expansion of Asian rice

Guangtong Gao[1,2,3], Maoxian Chen[1,2,3], Rong Mo[1,2,3], Nan Li[1,2], Yunzhang Xu[1,2,4], Yingqing Lu[1,2,5]

[1]State Key Laboratory of Systematic and Evolutionary Botany, Institute of Botany, Chinese Academy of Sciences, 20 Nan Xin Cun, Beijing 100093, China

[2]University of Chinese Academy of Sciences, Beijing 100049, China

[3]Authors of equal contribution

[4]College of Agriculture and Animal Husbandry, Qinghai University, Xining 810016, China.

[5]Author for correspondence

Figure S1 Additional alleles identified in independent surveys of Asian rice.

Figure S2 Identifications of background mutations in the genomic regions taken as references of alleles at an oscillator locus.

Figure S3 Approximate distribution of *OsPRR37* alleles surveyed in this study.

Table S1 Genomes analyzed in this study.

Table S2 Polymorphic sites at *OsCCA1* (Os08g06110.2) from the ten genomes.

Table S3 Polymorphic sites at *OsPRR95* (Os09g36220.1) from the ten genomes.

Table S4 Polymorphic sites at *OsPRR37* (Os07g49460.1) from the ten genomes.

Table S5 Polymorphic sites at *OsPRR59* (Os11g05930.1) from the ten genomes.

Table S6 Polymorphic sites at *OsPRR1* (Os02g40510.1) from the ten genomes.

Table S7 Additional alleles of *OsPRR1* detected in this study.

Table S8 Ten genomes of *A. thaliana* surveyed for alleles of oscillator genes in this study.

Table S9 Additional accessions of *O. sativa* surveyed in this study.

Table S10 Mutations identified at *AtCCA1* and checked against genome of *A. arenosa* (*Aa*).

Table S11 Mutations identified at *AtPRR9* and checked against genome of *A. arenosa* (*Aa*).

Table S12 Mutations identified at *AtPRR7* and checked against genome of *A. arenosa* (*Aa*).

Table S13 Mutations identified at *AtPRR1* and checked against genome of *A. arenosa* (*Aa*).

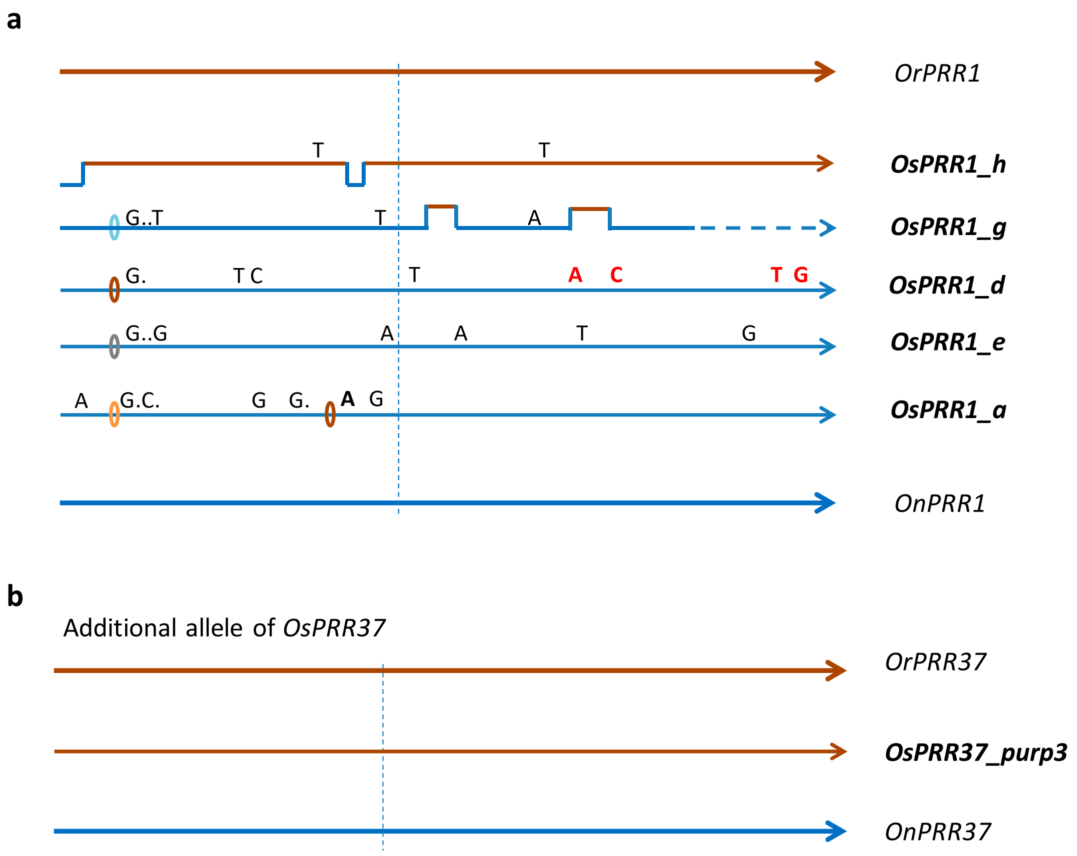

Figure S1 Additional alleles identified in independent surveys of Asian rice. **a** Five more alleles of *OsPRR1* identified in additional landraces/varieties. The format follows that of Figure 1. **b** *OsPRR37_purp3* identified in landrace Jixuenuo. It carries the same 5' and coding regions as those of *OrPRR37*.

a

Mutations in *OsPRR1*-reference sequences (3’ intergenic region, an alignment (TGA..GAG) of 875 bp)

Source	87–88	115–125	299	303	347–352	438	440	499	599	615	660	674	701	704	755	821
OrPRR1	GT	T.	-	G	C.	A	G	T	G	A	A	T	C	C	A	A
OnPRR1	GT	T.	C	G	C.	A	G	T	G	A	A	T	C	C	A	A
*OsPRR1_b-aus*N22	GT	T.	T	G	-	A	G	T	G	A	A	T	C	C	A	A
*OsPRR1_c-aus*Natel	-	T.	C	G	C.	A	G	T	T	C	G	T	A	C	A	A
OsPRR1_j-Napp	GT	T.	C	G	C.	A	G	T	G	A	A	T	C	C	A	A
OsPRR1_n-Kitaake	GT	-	C	A	C.	C	G	T	G	C	A	T	C	C	C	G
*OsPRR1_m-troj*Ketan	GT	T.	T	G	C.	A	G	C	G	A	A	T	C	C	A	A
*OsPRR1_i-troj*CM	GT	T.	C	G	C.	C	G	T	G	C	A	T	C	C	C	G
OsPRR1_f-aromatic	GT	T.	C	G	C.	A	G	T	G	A	A	A	C	C	A	A
Allelic split			1/2			1/2				1/3					1/2	1/2

Example:

Allele-source	Mutation number		
	5'	875 bp	cds
*OsPRR1_b-aus*N22	4.2	1.50	1
*OsPRR1_c-aus*Natel	4.2	4.33	2
OsPRR1_j-Napp	3.2	0.00	0
OsPRR1_n-Kitaake	3.2	3.88	1
*OsPRR1_m-troj*Ketan	3.2	1.50	2
*OsPRR1_i-troj*CM	4	1.88	0
OsPRR1_f-aromatic	6	1.00	2

Number of background mutations in all alleles: 14

b

Mutations in *OsCCA1*-reference sequences (intron region, an alignment (GT..AG) of 3963 bp)

Source	109	462	463	464	744	1221	1239	1356	1376	1429	1602	1610	1820	1871	1978	1985	1998	2334	2502	2565	2758	2953	3189	3206	3371	3608	3737
OrCCA1	A	-	-	A	C	C	G	T	C	C	A	T	-	G	C	G	C	T	G	T	C	C	T	A	T	C	G
OnCCA1	A	C	-	-	C	C	G	T	C	C	A	T	-	G	C	G	A	T	G	T	C	C	T	A	T	C	G
OsCCA1_e-aus	A	C	-	-	C	C	G	T	C	C	A	T	-	G	C	G	A	T	G	T	C	C	T	A	T	C	G
OsCCA1_b-9311,arom	A	-	-	A	C	C	G	T	C	C	T	T	-	G	C	A	A	A	G	T	C	C	T	A	T	T	G
OsCCA1_c-Shuhui	A	-	-	-	C	C	G	T	C	C	A	T	-	G	C	G	C	T	G	T	C	C	A	A	T	C	G
OsCCA1_d-Minghui	A	C	-	A	C	A	G	-	C	T	A	T	T	G	C	G	-	T	G	T	C	A	T	A	T	C	G
OsCCA1_a-Nipp	G	-	A	A	T	C	G	T	T	C	A	G	-	T	T	G	A	T	A	G	A	C	T	G	C	C	G
*OsCCA1_f-troj*Ketan	A	A	A	A	C	C	A	T	C	C	A	T	-	G	C	G	A	T	G	T	C	C	T	A	T	C	A
*OsCCA1_c-troj*CM	A	-	-	-	C	C	G	T	C	C	A	T	-	G	C	G	C	T	G	T	C	C	A	A	T	C	G

Allele-source	Mutation number		
	5'	Intron3	CDs
OsCCA1_e-aus	0	0	0
OsCCA1_b-9311,*aromatic*	0	4	2
OsCCA1_c-Shuhui	0	1	0
OsCCA1_d-Minghui	1.0	6	2
OsCCA1_a-Nipp	1.0	12	1
*OsCCA1_f-troj*Ketan	0	3	1
OsCCA1_c-trojCM	0	0	0

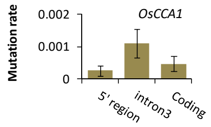

Number of background mutations in all alleles: 26

c

Mutations in *OsPRR37*-reference sequences (3’ intergenic region, an alignment (ATC..GCT) of 1035 bp)

Source	68	69	80	125	168	191	224	364	413	563	600	628	658	673	684	692	752	773	853	916
OrPRR37	T	T	G	G	C	G	G	T	G	C	C	C	G	G	G	-	-	C	G	A
OnPRR37	T	T	G	G	C	G	T	G	G	C	C	C	G	G	G	-	-	C	G	A
*OsPRR37_b-aus*N22	-	T	G	G	T	G	T	G	G	T	C	T	A	A	G	T	-	C	A	G
*OsPRR37_e-aus*Natel	-	T	G	G	C	G	T	A	G	C	T	T	G	G	G	-	-	T	G	G
OsPRR37_d-9311	-	T	G	G	C	G	T	G	G	T	C	T	A	A	G	T	-	C	A	G
OsPRR37_b-Shuhui	-	T	G	G	C	G	T	G	G	T	C	T	A	A	G	T	-	C	A	G
OsPRR37_i-Minghui	T	-	G	G	C	G	T	G	G	T	C	T	A	A	G	T	-	C	A	G
OsPRR37_a-Nipp	T	-	G	T	C	T	C	G	A	C	C	C	G	G	A	-	-	C	G	A
OsPRR37_h-Kitaake	-	T	G	T	C	T	C	G	A	C	C	C	G	G	A	-	-	C	G	A
*OsPRR37_f-troj*Ketan	-	T	A	G	C	G	G	T	G	C	C	C	G	G	G	-	-	C	G	A
*OsPRR37_c-troj*CM	-	T	G	G	C	G	T	G	G	T	C	T	A	A	G	T	-	C	A	G
OsPRR37_g-aromatic	-	T	G	G	C	G	T	A	G	C	T	T	G	G	G	-	G	T	G	G
Allelic split	1/6	1/2																		

Number of background mutations in all alleles: 20

d

Mutations in *OsPRR95*-reference sequences (3’ intergenic region, an alignment (GTG..GAGA) of 1037 bp)

Source	179	471	498	604	670	673-679	705	786	992
OrPRR95	-	-	A	G	C	A.	C	C	C
OnPRR95	-	-	A	G	C	A.	C	C	C
*OsPRR95_e-aus*N22	-	T	A	A	T	A.	C	C	C
*OsPRR95_f-aus*Natel	-	T	A	A	T	A.	C	C	C
OsPRR95_b-9311	-	T	A	G	C	-	T	C	T
OsPRR95_b’-Shuhui	-	-	A	G	C	-	T	C	T
OsPRR95_d’-Minghui	-	-	G	G	C	A.	C	T	C
OsPRR95_a-Nipp	-	-	G	G	C	A.	C	T	C
OsPRR95_g-Kitaake	-	-	G	G	C	A.	C	T	C
*OsPRR95_c-troj*Ketan	A	-	G	G	C	A.	C	T	C
*OsPRR95_d-troj*CM	-	-	G	G	C	A.	C	T	C
OsPRR95_g-aromatic	-	-	G	G	C	A.	C	T	C
Allelic split		1/3	1/4	1/2	1/2		1/2	1/4	1/2

Number of background mutations in all alleles: 9

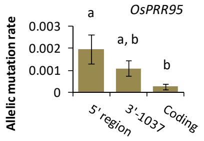

e

Mutations in *OsPRR59*-reference sequences (3’ intergenic region, an alignment (GTG..CTCT) of 1014 bp)

Source	180	458	460	507	712	795	829	859	870	925	945-951	979	985
OrPRR59	G	G	-	T	T	A	-	T	-	C	-	C	G
OnPRR59	G	G	-	T	T	A	-	T	-	C	-	C	G
*OsPRR59_a-aus*N22	G	G	A	T	T	A	-	T	-	C	-	C	G
*OsPRR59_f-aus*Natel	G	G	A	C	T	A	T	T	-	C	-	C	G
OsPRR59_b-9311	A	G	A	T	T	A	-	T	-	C	-	C	G
OsPRR59_c-Shuhui	G	A	-	T	A	A	-	C	T	A	6C	C	A
OsPRR59_e-Minghui	G	A	-	T	A	A	-	C	-	A	7C	C	A
OsPRR59_a-Nipp	G	G	A	T	T	A	-	T	-	C	-	C	G
OsPRR59_a-Kitaake	G	G	A	T	T	A	-	T	-	C	-	C	G
*OsPRR59_d-troj*Ketan	A	A	-	T	A	G	-	T	-	C	3C	C	G
*OsPRR59_a-troj*CM	G	G	A	T	T	A	-	T	-	C	-	T	G
OsPRR59_a-aromatic	G	G	A	T	T	A	-	T	-	C	-	C	G
Allelic split													

Number of background mutations in all alleles: 15

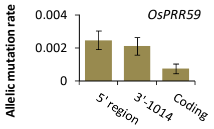

Figure S2 Identifications of background mutations in the genomic regions taken as references of alleles at an oscillator locus. **a** Mutations in the reference region of *OsPRR1*. Mutations (in red) are those absent in parental genomes and present in *Os* genomes only. Assignment of mutation is split among the shared mutations among allelic sequences (not in *aromatic*) when the emergence order of the alleles is uncertain, as shown in the last line of each table. Allelic mutation rate is shown in the graph below each table to summarize the result of comparisons. Those presented in Figure 1 are not included. **b** Mutations in the reference region of *OsCCA1*. **c** Mutations in the reference region of *OsPRR37*. **d** Mutations in the reference region of *OsPRR95*. Different letters indicate significant difference in means (one-tailed *t*-test, $P = 0.034$). **e** Mutations in the reference region of *OsPRR59*.

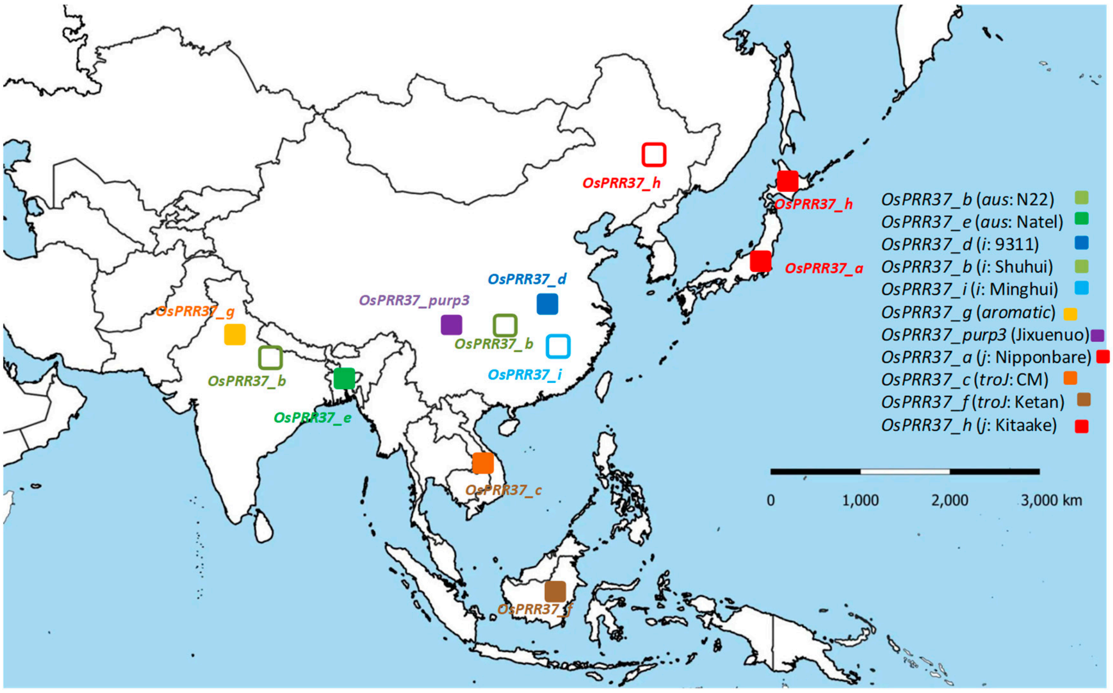

Figure S3 Approximate distribution of *OsPRR37* alleles surveyed in this study. The different alleles are marked in colors, with ones reported before shown in blank squares. The notation of allele follows that of Figure 7. The sequences of the alleles see Tables S4.

Table S1 Divergence of oscillator genes between orthologs of *O. rufipogon* and *O. nivara*.

Gene[a]	5'	Coding	Polymorphic sites[b]
OrCCA1 vs. *OnCCA1*	6	5	11
OrPRR95 vs. *OnPRR95*	9	8	17
OrPRR37 vs. *OnPRR37*	3	10	13
OrPRR59 vs. *OnPRR59*	13	6	19
OrPRR1 vs. *OnPRR1*	13	4	17

Note: [a] Comparisons did not include introns. [b] A cluster of continuously polymorphic nucleotides or indels is counted as one site for mutation.

Table S2 Polymorphic sites at *OsCCA1* (Os08g06110.2) from the ten genomes.

CCA1-5′ (1014 bp alignment, 1 mutation)

Source	99	204	477	640–655	658–659	884	968
OrCCA1	G	G	T	A.	--	T	C
OnCCA1	T	T	C	T.	TT	C	T
OsCCA1_e-aus	T	T	C	T.	TT	C	T
OsCCA1_d-Indica (Minghui)	T	T	C	T.	T-	C	T
OsCCA1_a-japonica	T	G	C	T.	--	C	C
*OsCCA1_f-troj*Ketan	T	T	C	T.	--	C	T
*OsCCA1_c-troj*CM (*i*: Shuhui)	G	G	T	A.	--	T	C
OsCCA1_b-aromatic (i: 9311)	G	G	C	A.	--	T	C

CCA1-cds (2160 bp alignment, 7 mutations)

Source	78	366	544	960	1252	1401	1457	1473	1488	1938	1957	2159
OrCCA1	T	T	C	C	A	C	C	C	G	T	G	G
OnCCA1	T	C	T	T	G	C	C	T	G	T	G	G
OsCCA1_e-aus	T	C	T	T	G	C	C	T	G	T	G	G
OsCCA1_d-Indica (Minghui)	G	T	T	T	G	T	C	C	G	C	G	G
OsCCA1_a-japonica	T	T	C	C	A	C	T	C	G	T	G	G
*OsCCA1_f-troj*Ketan	T	T	T	T	G	C	C	C	A	T	G	G
*OsCCA1_c-troj*CM (*i*: Shuhui)	T	T	C	C	A	C	C	C	G	T	G	G
OsCCA1_b-aromatic (i: 9311)	T	T	C	C	G	C	C	C	G	T	A	A

Note: An *Or* gene is in orange, *On* gene in blue, and *Os* mutations in grey, and only polymorphic sites between *Or* and *On* are colored. A nonsynonymous substitution is in red & parental one further underlined, assuming that *On mutation* is in a derived state relative to its ortholog *Or* gene. *troj* is for *tropical japonica,* and CM for variety Chao Meo. Allele shown in additional subgroups is indicated by the subgroup and variety (*i*: for subgroup *indica*). Minghui has a percentage of *aus* genome, thus classified as Indica for the subspecies.

Table S3 Polymorphic sites at *OsPRR95* (Os09g36220.1) from the ten genomes.

PRR95-5′ (1031 bp alignment, 16 mut)

Source[a]	62	80	179	278	282	284	285	287	289–293	294–309	413	673	748	787	883	928–929	930–932	947
OrPRR95	C	C	A	G	A	C	C	C	2C	12A	T	C	T	C	A	AG	-	G
OnPRR95	C	T	A	G	A	C	C	T	5C	14A	C	C	G	G	A	AG	GAG	T
OsPRR95_e-aus (N22)	A	T	A	A	C	C	G	C	2C	16A	C	C	G	G	A	AG	-	T
OsPRR95_f-aus (Natel)	A	T	A	A	C	-	G	C	2C	16A	C	C	G	G	A	AG	-	T
OsPRR95_b-indica (9311)	C	T	C	G	A	C	C	C	4C	10A	C	C	T	C	T	-	-	G
OsPRR95_b′-indica (Shuhui)	C	T	C	G	A	C	C	C	5C	11A	C	C	T	C	T	-	-	G
OsPRR95_a-japonica (Nipp)	C	C	A	G	A	C	C	C	-	15A	T	C	T	C	A	-	-	G
OsPRR95_g-japonica (Kitaake)	C	C	A	G	A	C	C	C	-	16A	T	A	T	C	A	-	-	G
OsPRR95_d′-indica (Minghui)	C	C	A	G	A	C	C	C	-	13A	T	C	T	C	A	-	-	G
OsPRR95_d- troj (CM)	C	C	A	G	A	C	C	C	-	12A	T	C	T	C	A	-	-	G
OsPRR95_c-troj (Ketan)	C	C	A	G	A	C	C	C	-	12A	T	C	T	C	A	-	-	G
OsPRR95_h-aromatic	C	C	A	G	A	C	C	C	-	15A	T	C	T	C	A	-	-	G

PRR95-cds (1872 bp alignment, 4 mutations)

Source	9	180	408	600	693	778	817	839	999	1057	1551	1833
OrPRR95	A	G	G	A	A	A	C	T	C	C	T	-
OnPRR95	A	A	G	C	C	T	T	C	C	A	G	-
OsPRR95_e-aus (N22)	A	G	G	A	C	T	T	C	C	A	G	-
OsPRR95_f-aus (Natel)	A	G	G	A	C	A	C	C	C	C	G	-
OsPRR95_b-indica (9311)	T	G	G	A	A	A	C	T	T	C	G	-
OsPRR95_b′-indica (Shuhui)	T	G	G	A	A	A	C	T	T	C	G	-
OsPRR95_a-japonica (Nipp)	T	G	G	A	A	A	C	T	T	C	G	-
OsPRR95_g-japonica (Kitaake)	T	G	G	A	A	A	C	T	T	C	G	-
OsPRR95_d′-indica (Minghui)	T	G	A	A	A	A	C	T	T	C	G	-
OsPRR95_d- troj (CM)	T	G	A	A	A	A	C	T	T	C	G	-
OsPRR95_c-troj (Ketan)	T	G	A	A	A	A	C	T	T	C	G	G
OsPRR95_h-aromatic	T	G	A	A	A	A	C	T	T	C	G	-

Note: [a] Subgroups are in italic and variety in parentheses. *troj* is for *tropical japonica,* and CM for variety Chao Meo. *Or* genes are in orange, *On* genes in blue, and single *Os* mutations in grey (shared one in purple or yellow).

Table S4 Polymorphic sites at *OsPRR37* (Os07g49460.1) from the ten genomes.

PRR37-5' (1036 bp alignment, 4 mutations)

Source	273	406-414	826-828	886	912
OrPRR37	T	C.	T--	A	C
OnPRR37	C	-	-	A	C
OsPRR37_b (*aus* (N22), *indica* (Shuhui))	C	-	TT-	G	A
OsPRR37_e (*aus* (Natel))	C	-	TT-	A	C
OsPRR37_d(i) (*indica* (9311,Minghui))	C	-	TT-	G	A
OsPRR37_a (h) (*japonica* (Nipp,Kitaake))	T	C.	T--	A	C
OsPRR37_f (*troj* (Ketan))	T	C.	T--	A	C
OsPRR37_c (*troj* (CM))	C	-	TT-	G	A
OsPRR37_g (*aromatic*)	C	-	TTT	A	C

PRR37-cds (2230 bp alignment, 15 mutations)

Source	47	125	140	204	506	641	664	667	881	888	904	1003	1056	1148	1222	1260	1267	1370	1386	1398	1516–1523	1968	2095	2130	2221
OrPRR37	C	T	C	G	G	A	G	G	A	-	A	A	C	G	T	G	C	A	T	G	G.	A	C	T	G.
OnPRR37	G	C	C	G	G	G	A	G	A	-	A	A	C	A	T	G	T	G	C	G	G.	G	C	T	T.
OsPRR37_b (*aus* (N22), *indica* (Shuhui))	G	C	C	G	G	G	G	G	A	-	A	A	C	A	T	A	T	G	C	T	G.	G	C	T	G.
OsPRR37_e (*aus* (Natel))	G	C	C	G	G	G	G	G	G	-	A	G	T	A	A	G	T	G	C	G	G.	G	T	T	G.
OsPRR37_i (*indica* (Minghui))	G	C	C	G	G	G	G	G	A	-	-	A	C	A	T	A	T	G	C	T	G.	G	C	T	G.
OsPRR37_d (*indica* (9311))	G	C	C	G	G	G	G	G	A	-	A	A	C	A	T	A	T	G	C	T	-	G	C	T	G.
OsPRR37_a (*japonica* (Nipp))	G	T	G	T	G	A	G	G	A	-	A	A	C	G	T	G	C	A	T	G	G.	G	C	T	G.
OsPRR37_h (*japonica* (Kitaake))	G	T	C	T	G	A	G	A	A	-	A	A	C	G	T	G	C	A	T	G	G.	G	C	C	G.
OsPRR37_f (*troj* (Ketan))	C	T	C	G	A	A	G	G	A	-	A	A	C	G	T	G	C	A	T	G	G.	A	C	T	G.
OsPRR37_c (*troj* (CM))	G	C	C	G	G	G	G	G	A	A	A	A	C	A	T	A	T	G	C	T	G.	G	C	T	G.
OsPRR37_g (*aromatic*)	G	C	C	G	G	G	G	G	A	-	A	G	T	A	A	G	T	G	C	G	G.	G	T	T	G.

Note: Annotation follows those of Table S2 and Table S3.

Table S5 Polymorphic sites at *OsPRR59* (Os11g05930.1) from the ten genomes.

PRR59-5' (1015 bp alignment, 14 mutations)

Source[a]	66	159–162	191	192	201	204	228–269	275	284–288	318–432	528	620	674–697	719	725	846	848	906	942	970	994–997	1003–1004
OrPRR59	C	-	G	T	A	A	*Or*	T	-	*Or*	A	G	T	-	A	A	-	T	A	C	C.	CT
OnPRR59	A	T.	G	C	A	T	*On*	C	T.	*On*	A	G	C	A	A	A	-	C	T	C	C.C	CT
OsPRR59_a (*jap, aus* (N22), *aro, troj* (CM))	C	-	G	T	A	A	Or-like	T	-	*Or*	G	G	T	-	A	A	-	T	T	C	C.	-
sPRR59_f (*aus* (Natel))	C	-	G	T	A	A	Or-like	T	-	*Or*	G	G	T.	-	A	A	-	T	T	T	C.	-
OsPRR59_b (*indica* (9311))	C	-	G	T	A	A	A.-C.	T	-	C.-T.	G	G	T.	-	A	A	-	T	T	C	C.	-
OsPRR59_c (*indica* (Shuhui))	C	T.	G	C	-	T	*On*	C	T.	*Or*	G	G	-	-	G	G	-	C	T	C	C.	-
OsPRR59_e (*indica* (Minghui))	C	T.	G	C	-	T	*On*	C	T.	*Or*	G	G	-	-	G	G	-	C	T	C	C.	-
OsPRR59_d (*troj* (Ketan))	C	T.	A	C	A	T	Or-like	T	T.	*Or*	A	C	C.	-	A	A	A	T	T	C	-	-

PRR59-cds (2034 bp alignment, 9 mutations)

Source	-54 – -1	36–38	54	276	324	380	528	693	792	1243	1335	1340	1566	1613	1858	2027
OrPRR59	-	-	C	C	G	C	G	A	C	C	C	G	T	C	A	G.
OnPRR59	-	G.	G	T	G	C	A	A	C	C	C	A	T	C	A	T.
OsPRR59_a(*jap, aus* (N22), *aro, troj* (CM))	-	G.	G	C	G	C	G	A	C	C	C	G	T	T	A	G.
OsPRR59_f (*aus* (Natel))	A.	G.	G	C	G	A	stop									
OsPRR59_b (*indica* (9311))	-	G.	G	C	G	C	G	A	C	C	C	G	T	T	A	G.
OsPRR59_c (*indica* (Shuhui))	-	-	C	C	G	C	G	A	C	C	C	G	T	C	A	T.
OsPRR59_e (*indica* (Minghui))	-	G.	G	T	A	C	A	A	T	C	T	A	-	C	stop	
OsPRR59_d (*troj* (Ketan))	-	G.	G	C	G	C	G	T	C	G	C	G	T	C	A	G.

Note: Annotation follows those of Table S2 and Table S3. Stop refers to the site for stop codon.

Table S6 Polymorphic sites at *OsPRR1* (Os02g40510.1) from the ten genomes.

PRR1-5' (1305 bp alignment, 28 mutations, un-alignable sites in number)

Source	19	39	86	88-100	101-103	104-125	126-534	541	549	554	590	611	623	650	667	671	673	687-696	838-841	842-848	856	876-880	962	982	1025	1101	1120	1207	1274	1286-1298	1303
OrPRR1	C	C	C	A.	A.	-	-	T	-	G	C	G	C	A	G	G	T	-	T.	T.	G	G.	C	C	G	G	T	C	G	G.	A
OnPRR1	-	T	G	A.	A.	GA.C	GA.C.	T	-	G	C	A	C	C	G	G	T	C.	T.	T.	G	-	C	C	G	G	C	C	-	-	G
OsPRR1_b (*aus* (N22), *indica* (Minghui))	-	C	G	A.	A.	4	T.	T	T	G	C	A	C	C	G	G	T	C.	T.	T.	T	-	C	C	A	G	C	C	-	-	G
OsPRR1_c (*aus* (Natel), *indica* (9311))	-	C	G	G.	-	5	GA.T.	T	-	G	C	A	C	C	G	G	T	C.	-	T.	G	-	C	C	A	G	C	C	-	-	G
OsPRR1_j (*japonica* (Nipp))	-	C	G	A.	A.	-	-	T	-	G	C	A	T	C	G	G	T	C.	T.	-	G	-	C	T	A	G	C	C	-	-	G
OsPRR1_n (*japonica* (Kitaake))	-	C	G	A.	A.	GG.	G. T.	T	-	C	C	A	C	C	G	G	T	C.	T.	T.	G	-	C	C	A	G	C	C	-	-	G
OsPRR1_m (*troj* (Ketan))	-	C	G	A.	A.	GT.	G. C.	C	-	G	C	A	C	C	G	G	T	C.	T.	T.	G	-	C	C	A	G	C	C	-	-	G
OsPRR1_i (*troj* (CM))	-	T	C	A.	-	15	C.	T	-	G	C	G	C	A	G	G	T	-	T.	T.	G	-	T	C	G	G	T	C	-	-	A
OsPRR1_f (*aromatic*)	-	C	G	A.	A.	GA.G	G.T.T.	T	-	G	G	A	C	C	A	G	T	C.	T.	T.	G	-	C	C	A	C	C	T	-	-	G

PRR1-cds (1557 bp alignment, 8 mutations)

Source	141	198	510	677	710	750	754	792	823	881	933	970	1113	1171	1299	1398	1474
OrPRR1	C	A	G	C	C	A	G	G	G	G	A	T	A	G	C	G	A
OnPRR1	C	G	G	T	C	G	G	A	G	G	A	T	A	G	C	G	A
OsPRR1_b (*aus* (N22), *indica* (Minghui))	C	G	A	T	C	G	G	A	G	G	A	T	A	G	C	G	A
OsPRR1_c (*aus* (Natel), *indica* (9311))	C	G	G	T	C	G	A	A	G	G	A	T	A	A	C	G	A
OsPRR1_j (*japonica* (Nipp))	C	A	G	T	C	G	G	A	G	G	A	T	A	G	C	G	A
OsPRR1_n (*japonica* (Kitaake))	C	A	G	C	C	A	G	A	G	G	A	T	C	G	C	G	A
OsPRR1_m (*troj* (Ketan))	C	G	G	T	T	G	G	A	G	G	A	A	A	G	C	G	A
OsPRR1_i (*troj* (CM))	C	A	G	C	C	A	G	G	G	G	A	T	A	G	C	G	A
OsPRR1_f (*aromatic*)	C	G	G	T	C	G	G	A	G	A	A	T	A	G	T	G	A

Note: Annotation follows those of Table S2 and Table S3.

Table S7. Additional accessions of *O. sativa* surveyed in this study.

Locus	Allele	ccessions (Plant II	Year	Sample size	Source	Location of source*
OsCCA1	*OsCCA1_a*	o6(3-6)	2018	6	Heidao	Heilongjiang
		p21-42(5-4)	2018		RIL298	-
		p21-42(6-5)	2018		RIL298	-
		P2-23(3-7)	2018		RIL315	-
		P2-23(4-3)	2018		RIL315	-
		P2-68(7-4)	2018		RIL315	-
	OsCCA1_c	o156(2-3)	2018	8	jixuenuo	Yunnan
		o85(2-6)	2018		Yunjing20	Yunnan
		P1-64(5-4)	2018		RIL315	-
		P2-24(2-4)	2018		RIL298	-
		P2-34(16-6)	2018		RIL298	-
		p24-75(7-3)	2018		RIL315	-
		p24-78(3-3)	2018		RIL315	-
		p24-78(4-3)	2018		RIL315	-
	OsCCA1_d	B16-44(10-8)	2018	14	RIL309	cross between indica and japonica
		B16-84(7-6)	2018		RIL309	-
		B17-11(5-6)	2018		RIL309	-
		B17-13(4-2)	2018		RIL309	-
		B17-13(10-2)	2018		RIL309	-
		B6-132(7-2)	2018		RIL301	cross within indica
		B6-132(8-3)	2018		RIL301	-
		B6-184(9-5)	2018		RIL301	-
		B6-184(11-4)	2018		RIL301	-
		P7-115(3-2)	2018		RIL298	cross between indica and japonica
		P7-215(7-3)	2018		RIL298	-
		P7-215(8-4)	2018		RIL298	-
		P14-102(2-6)	2018		RIL298	-
		P14-102(8-2)	2018		RIL298	-
OsPRR37	*OsPRR37_h (purp1)*	P2	2017	7	RIL315	H143
		P5	2016/2017		RIL315	-
		P9	2016		RIL298	-
		P12	2016		RIL298	-
		P14	2016		RIL298	-
		p7	2017		RIL298	-
		p14	2017		RIL298	-
	OsPRR37_b (purp2)	P1	2016/2017	10	RIL315	-
		P3	2017		RIL315	-
		P6	2016/2017		RIL298	-
		P10	2016		RIL298	-
		p7	2017		RIL298	-
		p13	2016/2017		RIL298	-
		p16	2016/2017		jixuenuo	Yunnan
		Z2	2016/2017		RIL315	-
		Z30	2016		RIL298	-
		B8	2016		RIL301	cross within indica
	OsPRR37_purp3	B11	2016	6	RIL309	cross between indica and japonica
		B12	2016		RIL309	-
		B13	2016		RIL309	-
		B15	2016		RIL309	-
		p7	2017		RIL298	-
		p14	2017		RIL298	-
OsPRR1	*OsPRR1_a*	P2-68(3-4)	2018	6	RIL315	-
		P2-68(3-6)	2018		RIL315	-
		A10	2017		Jiabala	Tibet
		A11	2017		Taizhong65	Taiwan
		A12	2017		Aituogu151	Sichuan
		A16	2017		Yue2	Guangdong
	OsPRR1_b	P7-115(2-2)	2018	5	RIL298	-
		P7-115(3-2)	2018		RIL298	-
		P14-102(2-6)	2018		RIL298	-
		B17-13(4-2)	2018		RIL309	-
		B17-13(10-2)	2018		RIL309	-
	OsPRR1_c	B6-132(7-2)	2018	6	RIL301	-
		B6-132(8-3)	2018		RIL301	-
		B6-184(9-5)	2018		RIL301	-
		B6-184(11-4)	2018		RIL301	-
		B17-11(5-2)	2018		RIL309	-
		B17-11(5-6)	2018		RIL309	-
	OsPRR1_d	P2-23(3-7)	2018	7	RIL315	-
		P2-23(4-3)	2018		RIL315	-
		P2-75(3-3)	2018		RIL315	-
		P2-75(6-4)	2018		RIL315	-
		p24-78(3-3)	2018		RIL315	-
		p24-78(4-3)	2018		RIL315	-
		o156	2018		jixuenuo	Yunnan
	OsPRR1_e	p21-42(5-4)	2018	5	RIL298	-
		p21-42(6-5)	2018		RIL298	-
		P7-215(7-3)	2018		RIL298	-
		P7-215(8-4)	2018		RIL298	-
		A03	2017		Longjin1	Jilin
	OsPRR1_g	P2-67	2018	2	RIL315	-
		P17-38	2018		RIL298	-
	OsPRR1_h	o85	2018	2	Yunjing20	Yunnan
		BJ346	2018		IR661	IRRI

Note: * Location for crossed progeny is not defined and indicated by "-".

Table S8. Additional alleles of *OsPRR1* detected in this study.

PRR1-5' (1306 bp alignment, 18 mutations)

Gene	19	39	57	86	88–100	101–103	104–124	125–533	542	555	592	612	624	651	670	672	674	688–697	707	838–841	843–849	855–856	857	881–885	963	965–976	983	985	1026	1102	1121	1137	1208	1244	1249	1274	1287–1299	1304
OrPRR1	C	C	C	C	A.	A-	-	-	T	G	C	G	C	A	G	G	T	-	A	C.	T.	T.	G	A.	C	C.	C	C	G	G	T	A	C	C	G	G	G.	A
OnPRR1	-	T	C	G	A.	A.	GA.G	G.C.C.	T	G	C	A	C	C	G	G	T	C.	A	C.	T.	T.	G	-	C	C.	C	C	G	G	C	A	C	C	G	-	-	G
OsPRR1_a (*i*: Zhengxian96)	-	T	A	G	A.	A.	GG.C	G.C.T.	T	G	C	A	C	C	G	G	T	C.	G	C.	T.	G.	G	-	C	-	C	C	A	G	C	G	C	C	G	-	-	G
OsPRR1_d (*i*: Jixuenuo)	-	T	C	G	A.	A.	GG.A.G.	G.T.C.	T	G	C	A	C	C	G	T	C	C.	A	C.	T.	T.	G	-	C	C.	C	C	G	G	C	A	C	C	G	-	-	G
OsPRR1_e (*j*: Heidao)	-	T	C	G	A.	A.	GG.G. A.	G..G.	T	G	C	A	C	C	G	G	T	C.	A	C.	T.	T.	G	-	C	C.	C	C	G	G	C	A	C	C	A	-	-	G
OsPRR1_g (*i*: Guangluai15)	-	T	C	G	A.	A.	GG.A.A.	G..TT.	T	G	C	A	C	C	G	G	T	C.	A	C.	T.	T.	G	-	C	C.	C	C	G	G	T	A	C	T	G	-	-	G
OsPRR1_h (*j*: Yunjing20)	-	C	C	C	A.	A-	-	-	T	G	C	G	C	A	G	G	T	-	A	C.	T.	T.	G	A.	T	C.	C	C	G	G	T	A	C	C	G	-	-	A

PRR1-coding regions (1557 bp alignment, 10 mutations)

Gene	141	198	445	510	549	660	677	710	747	750	754	792	823	881	933	970	1113	1171	1194	1299	1398	1474
OrPRR1	C	A	C	G	C	C	C	C	C	A	G	G	G	G	A	T	A	G	A	C	G	A
OnPRR1	C	G	C	G	C	C	T	C	C	G	G	A	G	G	A	T	A	G	A	C	G	A
OsPRR1_a (*i*: Zhengxian96)	C	G	C	G	C	C	T	C	C	G	G	A	G	G	A	T	A	G	A	C	G	A
OsPRR1_d (*i*: Jixuenuo)	T	G	C	G	C	C	T	C	C	G	G	A	A	G	C	T	A	G	A	C	T	G
OsPRR1_e (*j*: Heidao)	C	G	A	G	C	C	T	C	T	G	G	A	G	G	A	T	A	G	G	C	G	A
OsPRR1_g (*i*: Guangluai15)*	C	A	C	G	A	C	C	C	C	A	G	A	G	G	A							
OsPRR1_h (*j*: Yunjing20)	C	A	C	G	C	T	C	C	C	A	G	G	G	G	A	T	A	G	A	C	G	A

Note: * The coding region was not completely sequenced. Annotation follows those of Table S2 and Table S3.

Table S9. Ten genomes of *Arabidopsis thaliana* surveyed for alleles of oscillator genes in this study.

Source	Region	GenBank assembly	Genome coverage
Col	Poland	GCA_028009825.1	1000x
Ler	Germany	GCA_001651475.1	141x
C24	Iberia	GCA_000222345.1	75x
Kyo	Japan	GCA_902460305.1	61x
SALE	France	GCA_946408365.1	99x
ANGE	France	GCA_946415005.1	80x
MontM	France	GCA_946415625.1	84x
Rabacal	Portugal	GCA_946406895.1	97x
Cvi	Cape Verde	GCA_946414125.1	96x
Tanz	Tanzania	GCA_946409825.1	112x

Table S10. Mutations identified at *AtCCA1* and checked against that *of A. arenosa (Aa)*.

AtCCA1-5' region (1002 bp alignment)

Source	251[a]	252	274	280	292	355	357	450	610	670–671	841	921	942
Col	-	T	C	T	C	A	G	C	A	TT	G	G	G
Ler	-	T	C	T	C	A	G	C	A	TT	G	G	G
C24	-	T	C	T	C	A	G	T	A	TT	G	G	G
Kyo	-	T	C	T	C	A	G	C	A	TT	G	G	G
SALE	-	T	C	G	C	-	-	C	A	TT	G	G	C
ANGE	-	T	A	T	C	A	G	C	A	TT	G	G	G
MontM	-	T	C	T	C	A	G	C	A	TT	G	G	G
Rabacal	*A*	C	C	T	C	A	G	C	A	TT	T	G	G
Cvi	*A*	T	C	T	C	A	A	C	C	GC	G	A	G
Tanz	*A*	T	C	T	A	A	G	C	A	GC	G	G	G
AaCCA1-5'[b]	*?*	*?*	C	T	C	A	G	C	A	GC	G	G	G

AtCCA1 coding regions (1827 bp alignment)

Source	450[a]	858	981[c]	1485	1611	1641
Col	A	A	A	T	A	C
Ler	A	A	G	T	G	C
C24	A	A	A	T	A	C
Kyo	A	A	A	T	A	C
SALE	A	A	G	T	G	C
ANGE	A	A	A	T	A	C
MontM	A	A	A	T	A	C
Rabacal	A	A	G	T	G	C
Cvi	T	G	G	C	G	C
Tanz	T	A	G	T	G	T
AaCCA1-cds[b]	**?**	A	G	T	T	C

AtCCA1-3' (515 bp alignment(including 103 bp insert, C.), 412 bp without the insert)

Source	170	172-274	276-277	370-372	420	470-483	487	489	495
Col	t	-	TC	-	-	G.	-	A	C
Ler	t	-	TC	-	-	G.	-	A	C
C24	t	-	TC	-	-	G.	-	A	C
Kyo	t	-	TC	-	-	G.	-	A	C
SALE	t	-	TC	-	-	G.	-	G	C
ANGE	t	-	TC	-	-	G.	-	A	C
MontM	t	-	TC	-	-	G.	-	A	C
Rabacal	t	-	TC	-	-	G.	-	A	C
Cvi	t	-	TC	-	-	G.	-	A	C
Tanz	g	C.	CG	TTA	*A*	-	A	A	A
AaCCA1-3'[b]	G	C.	unalignable						

Note: [a] Site belonging to a string is in italic, suspected mutation in shade. [b] Site of *AaCCA1* not agreeing with mutation identification of *AtCCA1* is in orange, that agreeing with the assessment in blue, and uncertain site in question mark. [c] Site with polymorphic nucleotides higher than 30% in green and uncertain site in question mark. [d] Site showing nonsynonymous change is in red.

Table S11 Mutations identified at *AtPRR9* and checked against genome of *A. aerenosa (Aa).*

AtPRR9 - 5' region (1012 bp alignment, 8 mutations, 4 agreed by *Aa*)

Source	3[a]	35 – 38	208	219	314	373[b]	424	505	672	879	989
Col	T	TG.	T	G	T	*A*	A	G	G	G	C
Ler	T	TG.	T	G	T	*A*	A	G	G	G	C
C24	T	TG.	T	G	T	*A*	A	G	G	G	C
Kyo	T	TG.	T	G	T	*A*	A	G	G	G	C
SALE	T	TG.	T	G	T	-	T	G	T	A	T
ANGE	T	TG.	T	G	T	*A*	A	G	G	G	C
MontM	T	TG.	T	G	T	*A*	A	G	G	G	C
Rabacal	T	TG.	A	A	T	-	T	G	G	A	T
Cvi	C	TA-	A	G	C	-	A	G	G	A	T
Tanz	T	TG.	A	G	T	-	T	C	G	A	T
AaPRR9-5' [c]	C	TG.	A	A	?	A	-	G	G	A	T

AtPRR9 - coding regions (1407 bp alignment, 9 mutations, 8 agreed by *Aa*)

Source	45	73[d]	531	765	906	969	997	1133	1231	1317
Col	T	G	C	T	C	A	G	G	C	C
Ler	T	G	A	C	C	G	G	G	C	C
C24	T	G	C	T	C	A	G	G	C	C
Kyo	T	G	C	T	C	A	G	G	C	C
SALE	G	**C**	C	T	T	G	G	G	C	C
ANGE	T	G	C	T	C	A	G	G	C	C
MontM	T	G	C	T	C	A	G	G	C	C
Rabacal	G	G	C	T	C	A	T	G	C	T
Cvi	T	G	C	T	C	G	G	G	C	C
Tanz	G	G	C	T	C	G	G	A	G	C
AaPRR9-cds[b]	G	C	C	T	C	G	G	G	C	C

Note: [a] Suspected mutation is in shade. [b] Site with polymorphic nucleotides higher than 30% is in green and site in a string in italic. [c] Site unalignable is in question mark. Site of *AaPRR9* agreeing with mutation identification of *AtPRR9* is in blue and one disagreeing in orange. [d] Site showing nonsynonymous change is in red.

Table S12 Mutations identified at *AtPRR7* and checked against genome of *A. arenosa (Aa).*

AtPRR7-5' region (1018 bp alignment)

Source	79	81	117[a]	163–212[b]	267–272	288	311	323–324	329	330–340	344	369	384	431–447	512	527–528	557	575	581	585–594	622	634	649	668	779	787	995
Col	**A**	**T**	**G**	T.	**T.**	C	C	CT	C	TA	T	A	C	CC.	C	CT	T	G	C	C.	-	G	A	A	G	G	C
Ler	**T**	**C**	**-**	-T.	**-TG**	C	T	GC	T	CT	C	T	C	C.	C	-	A	A	C	T.	T	C	C	G	G	G	C
C24	**T**	**C**	**-**	T.	**-TG**	C	T	GC	T	CT	C	T	G	C.	C	-	A	A	C	T.	T	C	C	G	G	G	C
Kyo	**T**	**C**	-	T.	**-TG**	C	T	GC	T	CT	C	T	G	C.	C	-	A	A	C	T.	T	C	C	G	G	G	C
SALE	**A**	**T**	**G**	TC.	**T.**	C	C	CC	C	TA	T	A	C	CT.	C	CT	T	G	C	C.	-	G	A	A	G	G	G
ANGE	**A**	**T**	**G**	T.	**T.**	C	C	CT	C	TA	T	A	C	CC.	C	CT	T	G	C	C.	-	G	A	A	G	G	C
MontM	**T**	**C**	-	T.	**-TG**	C	T	GC	T	CT	C	T	C	C.	C	-	A	A	C	T.	T	T	C	G	G	G	C
Rabacal	**A**	**T**	**G**	T.	**T.**	G	C	CC	C	TA	T	A	C	C-	T	CT	T	G	T	C.	-	G	A	A	A	G	C
Cvi	**A**	**T**	**G**	T.	**T.**	C	C	CC	C	TA	T	A	C	CT.	C	CT	T	G	C	C.	-	G	A	A	G	A	C
Tanz	**A**	**T**	**G**	T.	**T.**	C	C	CC	C	TA	T	A	C	CT.	C	CT	T	G	C	C.	-	G	A	A	G	G	C
AaPRR7-5'[c]	**T**	**C**	**?**	T.	**?**	C	T	GC	C	TA	T	A	C	**C.**	C	CT	A	G	C	C.	-	G	-	A	G	T	C

AtPRR7 -coding regions (2184 bp alignment)

Source	187	258	261	279	450	631	884	997[d]	1025	1036	1037	1223	1485	1587	1774–1791	1806	1895	2113	2162	2168
Col	A	C	C	T	T	G	C	G	C	A	C	C	A	G	A.C.	G	T	C	T	A
Ler	**T**	C	T	T	T	G	C	A	T	A	T	C	A	G	C.T.	A	A	C	T	T
C24	**T**	C	T	T	C	G	C	A	T	A	T	C	A	G	C.T.	A	A	C	T	T
Kyo	T	C	T	T	C	G	C	A	T	A	T	C	A	G	C.T.	A	A	C	T	T
SALE	A	C	C	T	T	G	C	G	C	A	C	T	A	G	A.C.	G	T	C	G	A
ANGE	A	T	C	T	T	G	C	G	C	A	C	C	A	G	A.C.	G	T	C	T	A
MontM	**T**	C	T	C	T	G	C	G	T	A	T	C	G	G	C.T.	A	A	A	T	A
Rabacal	A	C	C	T	T	G	C	G	C	A	C	C	A	A	A.C.	G	T	C	T	A
Cvi	A	C	C	T	T	G	**T**	G	C	A	C	C	A	G	A.C.	G	T	C	T	A
Tanz	A	C	C	T	T	**A**	C	G	C	T	T	C	A	G	-	G	T	C	T	A
AaPRR7-cds[b]	A	C	C	T	?	G	C	G	C	A	C	T	A	G	A.C.	G	T	C	T	A

AtPRR7-3' regions (350 bp alignment)

**Source**	16[a]	66–77	142	285	333–347
Col	A	6TA	C	C	C.
Ler	A	5TA	C	C	C.
C24	A	5TA	C	C	C.
Kyo	A	5TA	C	C	C.
SALE	A	5TA	C	A	C.
ANGE	A	5TA	C	C	C.
MontM	A	5TA	C	C	C.
Rabacal	C	3TA	T	C	-

Cvi	C	3TA	T	C	C.
Tanz	C	3TA	T	C	C.
AaPRR7-3'[b]	A	?	C	?	C.

Note: [a] Site with polymorphic nucleotides higher than 30% is in green. [b] Suspected mutation is in shade. [c] Site of *AaPRR7* agreeing with mutation identification of *AtPRR7* is in blue and site unalignable in question mark. [d] Site showing nonsynonymous change is in red.

Table S13 Mutations identified at *AtPRR1* and checked against genome of *A. arenosa (Aa).*

AtPRR1 5' region (1020 bp alignment)

**Source**	**25**[a]	**71**[b]	88	**115**	**174**	**351**	**352**	**501**	503–504	505–506	**577**	**598**	704	**791**	992
Col	C	C	A	-	G	T	T	C	-	TC	T	A	A	A	G
Ler	T	T	A	A	G	-	-	C	-	-	T	A	A	G	G
C24	T	C	A	A	C	-	T	A	-	TC	T	A	G	A	T
Kyo	C	C	A	-	G	T	T	C	-	TC	T	A	A	G	G
SALE	T	C	A	A	C	-	T	C	-	TC	T	C	A	A	G
ANGE	C	C	A	-	G	T	T	C	-	TC	C	A	A	G	G
MontM	T	C	A	A	C	-	T	C	-	TC	T	C	A	A	G
Rabacal	C	C	A	A	G	-	T	C	-	TC	T	A	A	A	G
Cvi	T	C	-	A	C	-	-	C	-	-	T	A	A	G	G
Tanz	T	C	A	A	C	-	T	C	TC	TC	T	A	A	A	G
AaPRR1-5'[c]	?	C	A	A	C	?	T	C	?	TC	T	A	C	A	G

AtPRR1 coding regions (1857 bp alignment)

**Source**	**735**[d]	**1069**	**1106**	**1856**
Col	A	G	C	G
Ler	A	G	C	G
C24	T	A	C	G
Kyo	A	G	T	G
SALE	A	G	C	G
ANGE	A	G	T	G
MontM	A	G	C	G
Rabacal	A	G	C	A
Cvi	A	G	C	A
Tanz	A	G	C	G
AaPRR1-cds[b]	A	?	?	G

AtPRR1 3' regions (340 bp alignment)

Source	61[a]	109	319-320	321-332
Col	A	C	GT	T.
Ler	A	C	GT	T.
C24	A	C	GT	T.
Kyo	A	C	GT	T.
SALE	A	C	TC	-
ANGE	A	C	GT	T.
MontM	G	C	TC	-
Rabacal	A	C	GT	T.
Cvi	A	C	GT	T.
Tanz	A	A	TC	-
AaPRR1-3'[b]	A	C	GC	C.

Note: [a] Site with polymorphic nucleotides higher than 30% is in green. [b] Suspected mutation is in shade. [c] Site unalignable is in question mark. Site of *AaPRR1* agreeing with mutation identification of *AtPRR1* is in blue. [d] Site showing nonsynonymous change is in red.
